# Mechanistically informed circulating biomarkers are associated with acquired epilepsy after neonatal brain injury

**DOI:** 10.1186/s12974-026-03853-9

**Published:** 2026-05-09

**Authors:** Adam L. Numis, Renée A. Shellhaas, Janet S. Soul, Marisa A. Gardner, Courtney J. Wusthoff, Giulia M. Benedetti, Clara Di Germanio, Theo K. Bammler, David J. Erle, Walter L. Eckalbar, Charles E. McCulloch, Patrick J. Heagerty, Thomas R. Wood, Yvonne W. Wu, Sandra E. Juul, Daniel H. Lowenstein, Hannah C. Glass, the Neonatal Seizure Registry Study Group, Tayyba Anwar, Madison Berl, Catherine J. Chu, Linda S. Franck, Monica E. Lemmon, Betsy Thomas, Cameron Thomas, HEAL Consortium, Kaashif A. Ahmad, Sonia L. Bonifacio, Bryan A. Comstock, Fernando F. Gonzalez, Nathalie Maitre, Shavonne L. Massey, Dennis E. Maycock, Ulrike Mietzsch, Niranjana Natarajan, Gregory M. Sokol, Cameron Thomas, Krisa P. Van Meurs

**Affiliations:** 1 Department of Neurology and Weill Institute for Neurosciences, University of California San Francisco, San Francisco, CA 94143 USA; 2 Department of Pediatrics, University of California San Francisco, San Francisco, CA 94143 USA; 3 Department of Neurology, Washington University in St. Louis, St. Louis, MO 63110 USA; 4 Department of Neurology, Boston Children’s Hospital and Harvard Medical School, Boston, MA 02115 USA; 5 Department of Neurology, University of California Davis, Davis, CA 95616 USA; 6 Department of Pediatrics, Division of Pediatric Neurology, University of Michigan, Ann Arbor, MI 48108 USA; 7 Vitalant Research Institute, Core Immunology Laboratory, San Francisco, CA 94105 USA; 8 Department of Medicine, University of California, San Francisco, CA 94143 USA; 9 Department of Environmental and Occupational Health Sciences, University of Washington, Seattle, WA 98195 USA; 10 Department of Biostatistics, University of Washington, Seattle, WA 98195 USA; 11 Department of Epidemiology and Biostatistics, University of California San Francisco, San Francisco, CA 94143 USA; 12 Department of Pediatrics, University of Washington, Seattle, WA 98195 USA

**Keywords:** Epileptogenesis, Inflammation, Cytokines, MicroRNA, Neonatal Seizures, EEG, Epilepsy

## Abstract

**Background:**

Acute provoked neonatal seizures are a major risk factor for acquired epilepsy, yet clinicians lack reliable tools to identify neonates at highest risk. Preclinical data implicate innate immune activation and neuronal injury as key drivers of epileptogenesis, suggesting blood-based biomarkers could provide mechanistic insight and prognostic utility. We sought to identify biomarkers of epileptogenesis in neonates with acute provoked seizures after brain injury using multicenter cohorts.

**Methods:**

We conducted a prospective, multi-cohort analysis across two independent studies. *NSR-RISE* enrolled neonates with EEG-confirmed acute provoked seizures of diverse etiologies. The *HEAL* trial enrolled neonates with hypoxic-ischemic encephalopathy; analyses were limited to those with seizures. Plasma proteins were quantified 48—96 h after seizure onset. Associations with acquired epilepsy by 24-months were evaluated using log-link models with robust standard errors and false-discovery rate correction (FDR < 0.05). Significant proteins were added to models including established clinical predictors (≥ 3 days of EEG seizures and abnormal neurological examination at discharge). Exploratory pathway enrichment used KEGG databases. *NSR-RISE* participants also underwent plasma microRNA (miRNA) sequencing with integrative pathway analyses.

**Results:**

Among 35 neonates in *NSR-RISE*, 7 (20%) developed epilepsy; among 40 neonates in *HEAL*, 6 (15%) developed epilepsy. Across both cohorts, neonates with epilepsy had higher concentrations of the pro-inflammatory cytokine IL-1β and the neuronal injury marker UCHL1 compared to those without epilepsy. Growth hormone (GH), measured only in *NSR-RISE*, was decreased in neonates with epilepsy. Incorporation of biomarkers improved prognostic accuracy for epilepsy beyond clinical features alone (Area under the precision-recall curve (AUPRC) 0.30 (95%CI, 0.28—0.32) versus 0.91 (95%CI, 0.89—0.92); p < 0.001). Pathway enrichment analyses implicated innate immune signaling, including TLR/IL1/NF-κB-related and MAPK-associated IL-17 signaling. miRNA profiling identified 11 species differentially expressed between neonates with and without epilepsy, including brain-enriched miRNAs. Network analysis identified a co-expression module enriched for let-7f-5p and miR-146a-5p targeting TLR/IL1/NF-κB, MAPK, and JAK/STAT pathways.

**Conclusions:**

Across two cohorts, mechanistically informed biomarkers were associated with acquired epilepsy after neonatal seizures. IL-1β, UCHL1, and GH reflect inflammation, neuronal injury, and impaired trophic signaling, while circulating miRNAs provide complementary mechanistic insight. Findings support a translational biomarker panel and highlight inflammation as a biologically plausible therapeutic target.

**Supplementary Information:**

The online version contains supplementary material available at 10.1186/s12974-026-03853-9.

## Background

Seizures are a common sign of acute neurologic dysfunction in the neonatal period and affect more than 16,000 newborns in the United States every year [1]. Acute provoked neonatal seizures carry a substantial long-term burden beyond their immediate management challenges: approximately one in six affected neonates subsequently develops acquired epilepsy [2, 3]. Acquired epilepsy is defined by the emergence of recurrent unprovoked seizures after resolution of the initial acute symptomatic events and reflects a transition from injury-related seizures to persistent network hyperexcitability [4]. Despite this risk, clinicians lack reliable, generalizable tools to identify which neonates are most likely to develop epilepsy. Current prognostication primarily leverages clinical features, EEG, and MRI, yet these alone have limited sensitivity and fail to capture the biological processes that drive epileptogenesis in the injured neonatal brain [2, 3, 5, 6].

Preclinical studies suggest that innate immune activation is a key mechanism that links neonatal brain injury to subsequent epilepsy. After acute provoked seizures, microglial and astrocytic activation rapidly induce cytokine cascades, in particular IL-1β, IL-6, and TNF-α, with downstream engagement of TLR/IL-1/NF-κB and JAK/STAT signaling [7–9]. These pathways modulate neuronal excitability and synaptic remodeling, which subsequently lowers seizure threshold and promotes reorganization [10–13]. Importantly, these inflammatory molecules disrupt the integrity of an already compromised blood–brain barrier after injury, which increases permeability and allows measurement of brain-derived molecules in the peripheral circulation [14]. These inflammatory pathways are also targetable by commercially available therapeutics – for example, antagonism of IL-1 signaling reduces epileptogenesis in animal models [15–19]. Yet despite the biological plausibility and therapeutic tractability of these pathways, translational evidence that links circulating inflammatory signals to later epilepsy in neonates with acute provoked seizures remains limited.

MicroRNAs (miRNAs) provide a mechanistically distinct layer of regulation in the biology of epileptogenesis. As post-transcriptional regulators, miRNAs can rapidly tune inflammatory pathways following brain injury [20–25]. Expression of specific miRNAs, such as miR-146a and miR-203, correlates with activation of TLR/IL1/NF-κB and JAK/STAT signaling pathways in experimental epilepsy models and in human neuroinflammatory conditions [17, 26–28]. Circulating miRNAs are stable in plasma and enriched in CNS-derived species, offering a minimally invasive biomarker of brain pathology [29, 30]. However, pediatric data that links plasma miRNA signatures to later epilepsy, particularly following acute provoked neonatal seizures, remain underexplored.

Given these gaps, a mechanism-centric biomarker strategy is needed. Such a strategy would integrate (i) protein mediators of innate immune signaling, neuronal injury, and repair; (ii) regulatory miRNA networks, and (iii) pathway-level readouts of epileptogenesis. This approach could yield prognostic tools with biological interpretability and enable risk-stratified surveillance and early-phase interventional trials that target causal pathways.

To address this need, we leveraged two complementary multicenter cohorts to identify and validate prognostic biomarkers of acquired epilepsy after neonatal brain injury. The *Neonatal Seizure Registry—The Role of Inflammation after Neonatal Seizures and Later Development of Epilepsy* (*NSR-RISE*) study is a prospective cohort of neonates with EEG-confirmed acute provoked seizures of diverse etiologies, serving as a discovery platform for inflammatory and neuronal injury proteins as well as miRNA profiling with integrative pathway analyses. The *High-dose Erythropoietin for Asphyxia and EncephaLopathy* (*HEAL*) clinical trial was a randomized, double-blind, multicenter, placebo-controlled trial designed to test the efficacy of high dose erythropoietin for neuroprotection in term neonates with hypoxic-ischemic encephalopathy (HIE). For this analysis, we restricted *HEAL* inclusion to participants with documented acute provoked seizures, providing a validation cohort for protein biomarkers in *NSR-RISE* [31]. We focused on a shared biological window 48—96 h after acute provoked seizure onset, and hypothesized that circulating proteins and miRNAs measured in this subacute phase would predict the later development of acquired epilepsy and converge on common inflammatory pathways implicated in animal models of epileptogenesis.

## Materials and methods

### Study design and participants

The *NSR-RISE* study (Clinical Trial Number: NCT04259125; Registration Date: 02/04/2020) is a multicenter prospective cohort of term neonates ≥ 36 and ≤ 44 weeks gestational age with acute provoked neonatal seizures of diverse etiologies identified on continuous video-EEG (cEEG) across four US sites. We excluded neonates with suspected neonatal-onset epilepsy syndromes or concurrent diagnoses likely to independently cause adverse neurodevelopmental outcomes. Maternal demographics and neonatal course were abstracted from chart review and classified as previously described [2, 32, 33]. Participants’ families were contacted within one month of the children’s 1- and 2-year birthdays by telephone and a brief epilepsy questionnaire was administered that comprised standardized questions to evaluate for acquired epilepsy, seizure semiology, and use of anti-seizure medications [34–36]. Epilepsy status was confirmed through medical record review. Secondary outcomes included motor function (Gross Motor Function Classification System, GMFCS) and adaptive skills [WIDEA-FS: a brief, telephone assessment with good concurrent validity with the Bayley Scales of Infant and Toddler Development, third edition (BSID-III)—to characterize developmental outcomes] as previously described [32, 37].

**Fig. 1:**
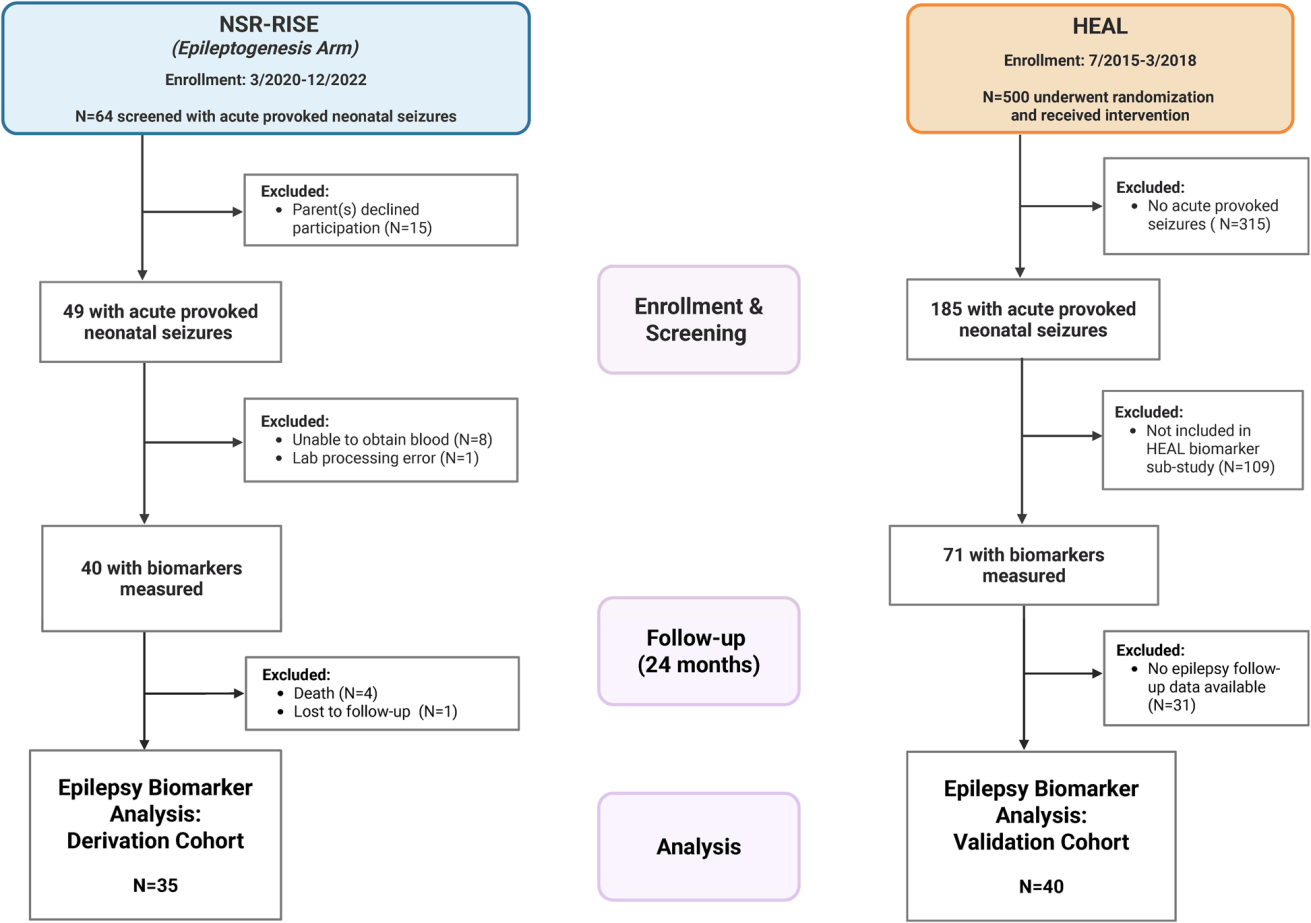
*NSR-RISE* and *HEAL* study flow diagram

The *HEAL* trial (Clinical Trial Number: NCT02811263; Registration Date: 6/17/2016) randomized 500 neonates with moderate-to-severe HIE to erythropoietin or placebo in addition to therapeutic hypothermia across 17 US sites [31]. Detailed screening, eligibility criteria, and exclusions prior to randomization have been previously reported in the primary trial publication [38]. In brief, eligibility required ≥ 36 weeks’ gestation, perinatal depression, moderate-to-severe encephalopathy by Sarnat criteria, and initiation of hypothermia within 6 h of birth, with exclusions including congenital or genetic anomalies, severe comorbidities, or anticipated loss to follow-up [38]. For the current analysis, we first identified participants within the randomized cohort with documented acute provoked neonatal seizures (Fig. 1). We then restricted the analysis to those within this seizure-defined group who were enrolled in the prespecified *HEAL* biomarker sub-study [39]. Longitudinal follow-up for interval medical history occurred at 4, 8, 12, 18, and 24 months of age and included assessment of GMFCS and the WIDEA-FS [40]. The *HEAL* trial also included an optional telephone questionnaire at each longitudinal follow-up timepoint to assess for interval diagnosis of acquired epilepsy and to detail outpatient anti-seizure medication prescriptions. Two authors (ALN and HCG) independently reviewed *HEAL* follow-up data to confirm epilepsy status with discrepancies adjudicated by consensus.

### Neonatal blood collection

In the *NSR-RISE* study, 1 mL blood samples were collected in EDTA tubes 48—96 h after the first cEEG-confirmed seizure. Within 1 h of collection, samples were centrifuged for 10 min at 1300g at 4°C. The supernatant was then centrifuged again at 16000g for 10 min to yield platelet-poor plasma, with aliquots frozen at -80 °C until analysis. In *HEAL*, blood was collected in EDTA tubes at three predefined intervals: [39] (1) baseline (median [IQR] 17.0 [12.9—21.8] hours after birth), (2) day 2, drawn 24 ± 2 h after the first study drug (placebo or erythropoietin) was given, and (3) day 4, 72 ± 2 h after the first study drug dose. Specimens were centrifuged at 2000g for 10 min and plasma aliquots stored at -80°C until analysis. For this study, we restricted analysis to day 4 samples to correspond to blood collection 48—96 h after acute provoked seizure onset [39, 41].

### Protein biomarker measurements

Thawed plasma was used for protein biomarker measurements. In HEAL, the 28 proteins measured have previously demonstrated adequate recovery rates in children with neurological disorders (Supplemental Table 1) [42]. In NSR-RISE, we measured all HEAL proteins, except for erythropoetin, and added five additional proteins to further interrogate epilepsy-associated inflammatory pathways (Supplemental Table 1): Growth Hormone (GH), Interleukin (IL)-4, IL-5, IL-21, and IL-23. We used the MILLIPLEX MAP Human High Sensitivity T Cell Panel from Millipore-Sigma to measure concentrations of (IL)-1, IL-6, IL-8, IL-10, IL-12p70, IL-13, IL-17A, macrophage inflammatory protein (MIP)-1α/chemokine ligand (CCL)3, MIP-1/CCL4, interferon (IFN)-γ, fractalkine/CX3CL1, and tumor necrosis factor (TNF)-α. We used the Luminex Multiplex assay (R&D Systems) to measure concentrations of putative biomarkers of neonatal brain injury and other growth factors involved in neuronal development and repair: brain-derived neurotrophic factor (BDNF), complement component (C5a), intercellular adhesion molecule 1 (ICAM-1)/cluster of differentiation (CD) 54, IL-1 receptor antagonist (IL-1RA), IL-33, monocyte chemoattractant protein 1 (MCP-1)/CCL2, neural cell adhesion molecule (NCAM-1)/CD56, neuroregulin-1—b—1 (NGB), neuron-specific enolase (NSE), S100 calcium-binding protein B (s100b), vascular cell adhesion molecule 1 (VCAM-1)/CD106, ubiquitin carboxy-terminal hydrolase-L1 (UCHL1), and vascular endothelial growth factor (VEGF). We measured glial fibrillary acidic protein (GFAP) and Tau with the R-PLEX assays (Meso Scale Diagnostics). In HEAL only, erythropoietin concentrations were measured with the Human Erythropoietin/EPO Quantikine ELISA Kit (R&D Systems). All assays followed manufacturer protocols, were run in duplicate, and results were averaged.

### miRNA biomarker isolation, library preparation, and sequencing

In *NSR-RISE*, miRNA analyses were performed in a subset of participants, including all neonates who developed acquired epilepsy and a 2:1 etiology-matched group of neonates with acute provoked seizures who did not develop epilepsy. Matching by seizure etiology was performed to minimize potential confounding from underlying brain injury mechanisms. We extracted total RNA from 200 μL of thawed plasma with the Plasma/Serum Circulating RNA Purification Kit (Norgen Biotek Corp., Thorold, ON, Canada) and followed manufacturer’s instructions. We prepared small RNA libraries with the NEBNext Multiplex Small RNA Library Prep Set for Illumina (catalog #E7300, New England Biolabs, Ipswich, MA) according to the manufacturer’s instructions, as previously described [43, 44]. Libraries were sequenced on a NovaSeq X PE50 (Illumina, San Diego, CA). The small RNA library preparation approach was selected to enable comprehensive, unbiased profiling of circulating miRNAs in plasma. This platform captures a broad range of miRNA species, including those implicated in inflammatory signaling, neuronal injury, and neurodevelopmental processes, rather than targeting a predefined subset. This design supports hypothesis-generating analyses aimed at identifying miRNA biomarkers and regulatory networks associated with epileptogenesis.

We assessed raw sequencing data with FastQC software (Babraham Bioinformatics, Cambridge, UK) [44]. We trimmed reads to remove adapters and low-quality bases; reads < 18 nucleotides were discarded. Processed files were uploaded to the Genboree Workbench (Bioinformatics Research Laboratory, Houston, Texas, version 4.6.3) for alignment using exceRpt to the human GRCh38 genome (Ensembl, Hinxton, UK). We removed reads mapping to UniVec and rRNA sequences before aligning to miRBase v22 (Manchester, UK) [45]. Expression values were normalized as reads per million (RPM).

### Statistical analysis

Analyses were conducted in R (version 4.0.2) [46]. Manhattan plots, volcano plots, receiver operator curves, and weighted gene co-expression networks (WGCNA) figures were generated in R. Bubble plots were generated in GraphPad Prism (version 10.3.1). Sankey plots were generated in Python (version 3.10).

### Power calculations

Measurements of IL-1β in neonatal blood spots are significantly correlated with plasma measurements, [47] and support the use of blood spot data as a reliable surrogate for plasma concentrations in power calculations for this study. Our preliminary data, where IL-1 concentrations were measured in neonatal blood spots, demonstrated that participants who developed acquired epilepsy after acute provoked neonatal seizures had a mean concentration of 101.1 pg/mL (standard deviation (SD) 10.3) and participants without epilepsy had a mean IL-1 of 77.2 pg/mL (SD 43.2) [48]. Power analysis indicated that with a 17% incidence of acquired epilepsy, [3, 33] 34 participants would provide 80% power to detect a group difference at α = 0.05. Additional proteins measured in this study were a priori deemed exploratory and were assessed at a false-discovery rate (FDR) < 0.05 (Benjamini-Hochberg) [49]. We conservatively accounted for a 18% attrition rate and prospectively targeted enrollment of 40 neonates in *NSR-RISE* [33]. In the previously completed *HEAL* trial, after excluding neonates without documented acute provoked neonatal seizures, we required a minimum of six participants with acquired epilepsy by 24 months of age to enable validation of the *NSR-RISE* results.

For miRNA analyses, all species were analyzed exploratorily, with statistical significance defined as an FDR < 0.05 and an absolute ≥ fourfold difference in expression between children with and without acquired epilepsy. We hypothesized upregulation of miR-146a and related IL-1β-pathway miRNAs in neonates who developed acquired epilepsy compared to those who remained seizure free. In adults with epilepsy, plasma miR-146a expression was increased 3.76-fold compared to adults without epilepsy, with an area under the receiver operator curve (AUC) of 0.77 (SD 0.049) [50, 51]. Based on these data and our planned sample size, our study was powered (80% at a two-sided α = 0.05) to detect an effect size (Cohen’s d) of 1.17, corresponding to an AUC of 0.80 and an estimated 1.5–2.25-fold change assuming a log₂ SD of 0.5—1.0, sufficient to detect expression differences of the magnitude previously observed for miR-146a and consistent with our predefined exploratory significance threshold (FDR < 0.05 and ≥ fourfold difference).

### Clinical and protein data

We used descriptive statistics to summarize baseline demographics and maternal/infant characteristics. Continuous variables were compared with ANOVA or Kruskal–Wallis tests, categorical variables with χ2 or Fisher exact tests. Protein distributions were summarized as medians, IQRs, and boxplots on a log_2_-scale. Due to minimal missingness, complete-case analyses were performed. Log-link regression with robust standard errors modeled the relationship between log₂-transformed biomarker concentrations and epilepsy, yielding relative risks (RRs) that reflect the change in epilepsy risk per doubling of biomarker concentration. IL-1β was the prespecified primary analyte and was evaluated at a nominal threshold of p < 0.05; all other proteins were considered exploratory and assessed at FDR < 0.05.

### Model evaluation with receiver operating characteristic (ROC) and precision-recall curves

Based on prior *NSR* cohorts, the most robust clinical predictors of acquired epilepsy in term neonates were ≥ 3 days of EEG-confirmed seizures and an abnormal neurological examination at discharge from the neonatal admission, after adjustment for relevant covariates [33]. These two variables were used as the foundation for model development, serving as the baseline clinical model. As these variables were not available in the *HEAL* dataset, all model evaluation was performed in the *NSR-RISE* cohort only. Given the small sample size, logistic regression models were restricted to a maximum of two covariates to minimize overfitting and ensure stable parameter estimation. This study was powered to detect differences in IL-1β, therefore this cytokine was the only biomarker prespecified for addition to the clinical model. We evaluated whether adding IL-1β to each of the two clinical predictors (EEG seizures ≥ 3 days + IL-1β; abnormal exam + IL-1β) improved discrimination compared with the clinical model alone. In exploratory analyses, additional candidate biomarkers that met an FDR < 0.05 across cohorts were added to IL-1β without clinical variables in order to assess potential synergistic molecular effects, acknowledging the likelihood of overfitting given the small number of events. Logistic regression models were trained using repeated, stratified fivefold cross-validation (100 repeats) to estimate out-of-sample performance. Model discrimination was evaluated by the AUC. Mean AUCs and 95% CIs were calculated from pooled out-of-fold predictions. Between-model comparisons used the DeLong test with the Sun-Xu algorithm [52, 53]. ROC curves were plotted from pooled predictions.

As we anticipated acquired epilepsy would be diagnosed in only 17% of neonates with acute provoked seizures followed through 24 months of age, we planned for an imbalanced dataset, with substantially fewer positive (epilepsy) than negative (no epilepsy) cases. In such settings, classification metrics such as AUC can be inflated by the predominance of the majority class, as a model may perform well simply by predicting the more frequent outcome. The area under the precision-recall curve (AUPRC) provides a more informative measure of model performance under class imbalance because it emphasizes the model’s ability to correctly identify true positive cases (precision) among all predicted positives (recall) [54]. Thus, AUPRC better reflects performance for acquired epilepsy in this study. However, AUC was also calculated to facilitate comparison with prior studies and because it remains the most widely used and easily interpretable measure of model discrimination in clinical research. Precision-recall models incorporating clinical variables and protein biomarkers were developed using the same strategy for AUC analyses with stratified fivefold cross-validation (100 repeats). AUPRCs were averaged across resamples with 95% CIs estimated by stratified bootstrap (B = 10,000). Between-model AUPRCs were compared using a paired permutation test based on within-subject prediction swapping with 10,000 resamples, [55] and empirical precision–recall curves were generated from pooled predictions.

### Protein pathway analyses

Protein enrichment analyses employed pathfindR (v2.4.2) [56]. For each protein biomarker, we used the ratio of the median concentration (acquired epilepsy versus no epilepsy), together with the unadjusted regression p-values as inputs. Proteins mapped to genes. Active subnetwork enrichment analysis was performed with the KEGG protein–protein interaction network with default parameters. Pathway enrichment of subnetwork genes was tested with one-sided hypergeometric tests. Outputs were significantly enriched pathways annotated with enrichment scores and FDR-adjusted p-values. Given the targeted nature of the inflammatory protein panel, pathway analyses were performed to provide biological context and should be interpreted as exploratory and hypothesis-generating rather than definitive evidence of pathway activation.

### miRNA differential expression and pathway analyses

Differential expression analysis was performed with DESeq2 (Bioconductor, version 1.48.2) [44, 57]. Results were corrected for multiple comparisons with Benjamini-Hochberg (FDR < 0.05). Differentially expressed miRNAs were mapped to predicted mRNA targets and analyzed with the QIAGEN Ingenuity Pathway Analysis (IPA) software to identify enriched canonical pathways [58]. miRNAs and expression values were mapped to the QIAGEN Knowledge Base. Networks were algorithmically generated based on molecular connectivity. Canonical pathway analysis identified the most significantly enriched pathways, with significance assessed by right-tailed Fisher’s exact test and expressed as the ratio of observed-to-expected molecules.

We also performed WGCNA on the miRNA expression data. WGCNA is a systems biology method that identifies clusters (modules) of highly correlated genes and then relates them to traits [59]. Raw miRNA data were transposed to obtain a samples × miRNA matrix and annotated with miRBase v22 [45]. After quality control, a signed co-expression network was constructed. An adjacency matrix was generated, converted to a topological overlap matrix (TOM), and miRNAs were hierarchically clustered based on TOM dissimilarity. Modules were identified by dynamic tree cutting (minimum size 50). Module eigengenes (first principal component) were calculated, and modules with eigengene correlation > 0.75 were merged. Epilepsy outcome was then matched to the expression dataset. Module-trait associations were tested by Pearson correlation with Student asymptotic p-values, and heatmaps were generated. For epilepsy-associated modules, intramodular connectivity was defined as the correlation of each miRNA with the module eigengene (module membership, MM). Gene significance (GS) was defined as the correlation between miRNA expression and epilepsy outcome. Hub miRNAs, considered candidate drivers of a phenotype, were defined as MM = > 0.90 and GS p < 0.05, as previously described [30, 60]. Hub miRNAs were mapped to predicted mRNA targets and analyzed in IPA to identify enriched canonical pathways.

### Ethics and reporting

The University of California, San Francisco Institutional Review Board (IRB) served as the central IRB for *NSR-RISE*. Written informed consent was obtained from at least one parent. Details of the *HEAL* trial consent process have previously been reported [31]. *NSR-RISE* is reported in accordance with STROBE [61]. *HEAL* was reported in accordance with CONSORT [62].

### Data availability

Data are available from the corresponding author upon request, subject to approvals. De-identified *NSR-RISE* and *HEAL* data are shared per NIH and institutional policies. Analytic code is available on GitHub at https://github.com/aln142/NSR-RISE.

## Results

### Study participants: *NSR-RISE* cohort

We screened 64 neonates with EEG-confirmed acute provoked seizures across participating sites. Fifteen (23%) parent(s) did not provide consent for blood collection and/or 24 months of longitudinal follow-up for assessment of epilepsy (Fig. 1). Among the 49 enrolled neonates, nine (18%) were excluded from the primary analysis due to inability to obtain blood or laboratory processing errors, resulting in 40 neonates with samples collected 48—96 h after seizure onset. Follow-up data were available for 35 of 40 (88%) neonates; four died prior to 24 months of age, and one lacked follow-up data. Seizure etiologies included HIE in 18 (51%), ischemic stroke in 7 (20%), intracranial hemorrhage in 5 (14%), meningitis/encephalitis in 3 (9%), and hypoglycemia with brain injury in 2 (6%). All neonates underwent MRI, with abnormalities present and imaging confirming the underlying etiology in all cases. Among the 35 neonates with outcome data, seven (20%) developed acquired epilepsy, with a mean age of epilepsy onset of 15.1 months (range: 2.7—21.6). No maternal or perinatal risk factors were associated with acquired epilepsy (Table 1). Neonates who developed epilepsy were more likely to have ≥ 3 days of EEG-confirmed seizures compared to those without epilepsy (*p* = 0.04) and showed a trend toward abnormal neurologic discharge examinations (*p* = 0.06), consistent with prior *NSR* results (Table 2) [3, 33].

**Table 1:** Characteristics of infants who survived acute provoked neonatal seizures with longitudinal epilepsy follow-up

Characteristic	*NSR-RISE* Cohort			*HEAL* Cohort			Between Cohort *p*-value
	Total	Epilepsy	No Epilepsy	Total	Epilepsy	No Epilepsy	
	*N* = 35	*N* = 7	*N* = 28	*N* = 40	*N* = 6	*N* = 34	
Maternal characteristics							
Race, n (%)							
White	18 (51)	3 (43)	15 (54)	31 (78)	4 (67)	27 (79)	0.045
Black	1 (3)	1 (14)	0	5 (13)	1 (17)	4 (12)	
Asian	2 (6)	0	2 (7)	0	0	0	
Multiple/other/unknown	14 (40)	3 (43)	11 (39)	4 (10)	1 (17)	3 (9)	
Hispanic ethnicity, n (%)	3 (9)	0	3 (11)	9 (23)	1 (17)	8 (24)	0.12
Education, ≤ high school, n(%)	2 (6)	1 (20)	1 (4)	15 (38)	2 (33)	13 (38)	< 0.001
Outborn delivery, n (%)	24 (69)	6 (86)	18 (64)	38 (95)	6 (100)	32 (94)	0.005
Maternal chorioamnionitis, n (%)	3 (9)	1 (17)	2 (8)	4 (10)	0	4 (12)	1.0
Cesarean delivery, n (%)	25 (71)	6 (86)	19 (68)	33 (83)	5 (83)	28 (82)	0.28
Neonatal Characteristics							
Gestational age, weeks, mean (SD)	39.1 (2.1)	38.8 (0.8)	39.2 (2.3)	38.9 (1.4)	38.9 (2.0)	38.9 (1.3)	0.63
Birth weight, kg, mean (SD)	3.2 (0.7)	2.9 (0.7)	3.3 (0.6)	3.3 (0.6)	2.9 (0.6)	3.4 (0.5)	0.51
Female, n (%)	11 (31)	2 (29)	9 (32)	18 (45)	1 (17)	17 (50.0)	0.25
5-min Apgar, median [IQR]	9 [6, 10]	8 [5, 10]	9 [6, 10]	2 [1, 4]	2 [1, 3]	2 [1, 4]	< 0.001
10-min Apgar, median [IQR]	9 [9, 10]	9 [8, 10]	9 [9, 10]	4 [3, 5]	5 [3.0, 6.0]	4 [3, 5]	< 0.001
Neonatal Clinical Course							
Therapeutic hypothermia, n (%)	11 (31)	2 (29)	9 (32)	40 (100)	6 (100)	34 (100)	< 0.001
Abnormal discharge exam, n (%)^b^	14 (40)	5 (71)	9 (32)	-	-	-	-
All oral feeding at discharge, n (%)	22 (63)	3 (43)	19 (70)	24 (62)	2 (40)	22 (65)	0.82
2-year Outcomes							
CP (GMCSF ≥ 1), n (%)	10 (29)^***^	6 (86)	4 (14)	13 (32)	3 (50)	10 (29)	0.80
Total WIDEA, mean (SD)	146 (37)^***^	83 (31)	161 (17)	139 (45)^*^	100 (43)	146 (42)	0.46
Receiving Services, n (%)^c^	13 (37)	5 (71)	8 (29)	27 (68)	5 (83)	22 (66)	0.01

All other *p*-values are ns (not significant). Significant associations within a study cohort are bolded. Significant associations between cohorts are identified in the last column

*Abbreviation CP* Cerebral palsy, *GMCSF* Gross motor function classification system, *IQR* Interquartile range, *WIDEA* Warner Initial Developmental Evaluation of Adaptive and Functional Skills—Full Scale

^a^Confirmed by continuous video-EEG in the *NSR-RISE* cohort and determined by chart review in the *HEAL* cohort

^b^The discharge neurological examination was defined as abnormal if consciousness, reflexes, or tone was clearly documented as abnormal in the medical record within 3 days of hospital discharge

^c^Including physical therapy, occupational therapy, speech and language therapy, vision therapy, and/or early intervention

^*^p < 0.05, ^**^p < 0.001, ^***^p < 0.001

**Table 2:** Seizure characteristics of infants who survived acute provoked neonatal seizures enrolled in *NSR-RISE*

**Characteristic**	***NSR-RISE*** ** Epilepsy Analysis Cohort**			
	Total	Epilepsy	No Epilepsy	p-value
	*N*=35	*N*=7	*N*=28	
Seizure etiology, n (%)				0.58
HIE	18 (51)	4 (57)	14 (50)	
Ischemic stroke	7 (20)	1 (14)	6 (21)	
Intracranial hemorrhage	5 (14)	0	5 (18)	
Meningitis/Encephalitis	3 (9)	1 (14)	2 (7)	
Other^a^	2 (6)	1 (14)	1 (4)	
Worst EEG Background during the 1^st^ 24 hours at study center^b^				0.20
Severely abnormal	12 (34)	4 (57)	8 (29)	
EEG seizure frequency at the study center, n (%)				0.77
Few (<7)	12 (34)	1 (14)	11 (39)	
Many isolated/frequent (≥7)	15 (43)	4 (28)	11 (18)	
Status epilepticus	8 (23)	2 (29)	6 (21)	
Three or more days of seizures, n (%)	6 (17)	3 (43)	3 (11)	0.04
Initial ASM Load, n (%)				0.56
Phenobarbital	30 (86)	7 (100)	23 (89)	
Fosphenytoin	0	0	0	
Levetiracetam	2 (6)	0	2 (8)	
Other	3 (9)	0	3 (11)	
Incomplete response to initial loading dose of ASM, n (%)	25 (71)	6 (86)	19 (68)	0.88

*Abbreviations ASM* Anti-seizure medication, *HIE* Hypoxic-ischemic encephalopathy

^a^Hypoglycemia with brain injury and uncategorized

^b^Severely abnormal was defined as burst-suppression, flat trace, depressed and undifferentiated, or electrocerebral inactivity. Mildly or moderately abnormal was defined as excessive negative sharp waves, positive sharp waves, excessive discontinuity, or asynchrony

### Study participants: *HEAL* cohort

Among 500 participants in the *HEAL* trial that underwent randomization and received intervention, 185 (37%) had documented acute provoked neonatal seizures (Fig. 1). Of these, 71 (38%) had been included in a predefined biomarker sub-study [39]. Among these 71 neonates, 40 (56%) had caregiver-reported epilepsy outcomes, and six (15%) developed acquired epilepsy. Erythropoietin exposure did not differ significantly between neonates with and without epilepsy (67% vs 38%, *p* = 0.40). Neonatal characteristics and 24-month outcomes data varied by availability of epilepsy follow-up data (Supplemental Table 2). Neonates whose families responded to epilepsy follow-up questions had less severe neonatal encephalopathy than non-responders (35% vs 65%, *p* = 0.03), yet through 24 months of age responding families’ children had lower WIDEA scores (139 vs 177, *p* = 0.03), higher rates of cerebral palsy (32% vs 0%, *p* = 0.001), and greater use of developmental services (68% vs 29%, *p* < 0.03), consistent with more severe post-discharge morbidity.

### Between cohort differences

Differences in maternal and neonatal characteristics between neonates enrolled in *NSR-RISE* versus *HEAL* reflected differences in study design and recruitment center demographics (Table 1). Both studies collected samples within the prespecified 48—96 h window after seizure onset, supporting valid cross-cohort comparisons despite modest differences in timing. In *NSR-RISE*, blood was collected a median 60 h after seizure onset (IQR 51—83). In a subset of 14 of 40 (35%) *HEAL* patients included in this analysis who had cEEG reviewed centrally by two neurophysiologists for seizure burden, [41] day 4 blood samples were collected at a median 79 h after seizure onset (IQR 72—80; p = 0.003). This variation was attributable to protocol-specified collection timepoints in the *HEAL* trial rather than differences in clinical course or sampling procedures.

### Protein biomarker analyses

**Fig. 2:**
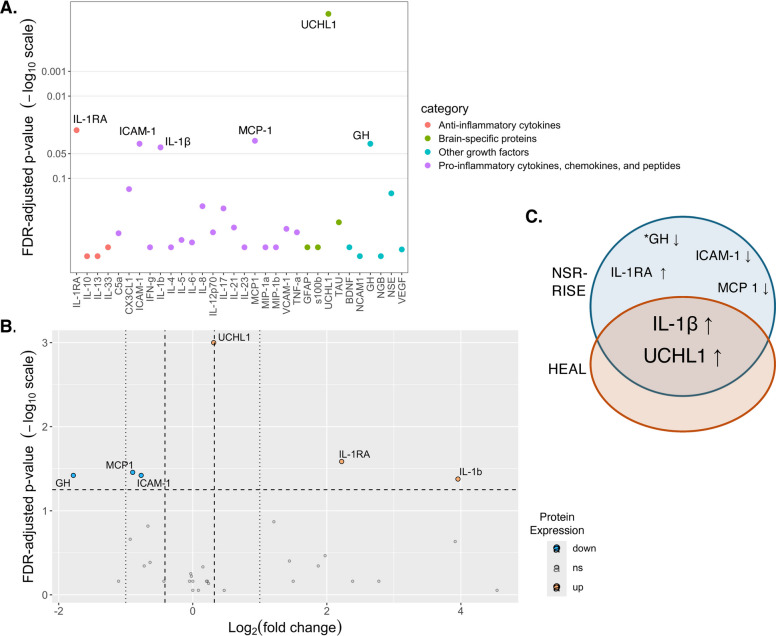
Differential expression of biomarker proteins across cohorts. **A** FDR-adjusted p-values for plasma protein biomarkers grouped by category (anti-inflammatory cytokines, brain-specific proteins, other growth factors, and pro-inflammatory cytokines, chemokines, and peptides) in the *NSR-RISE* cohort. **B** Volcano plot of log2(fold change) versus –log10(p value) highlighting direction and magnitude of protein expression differences in the *NSR-RISE* cohort. The dashed horizontal reference line is at an FDR adjusted p-value of 0.05. The vertical dashed line represents a 25% change in protein expression between neonates with and without acquired epilepsy after acute provoked neonatal seizures; the dotted vertical line represents a 100% change in protein expression. **C** Venn diagram summarizing proteins with significant expression changes in *NSR-RISE* and *HEAL* cohorts

In *NSR-RISE*, 32 protein biomarkers were measured (Supplemental Table 1). In addition to IL-1β, seven proteins showed nominal associations with acquired epilepsy (IL-1RA, CX3CL1, GH, ICAM-1, MCP1, NSE, UCHL1), and five of seven remained significant after FDR adjustment (Fig. 2A). Neonates with acquired epilepsy had higher concentrations of IL-1β (RR 1.5, 95% CI: 1.01—2.3), IL-1RA (RR 1.7, 95% CI: 1.2—2.3), and UCHL1 (RR 3.2, 95% CI: 2.2—5.2), and lower concentrations of ICAM-1 (RR 0.34, 95% CI: 0.2—0.7), MCP-1 (RR 0.72, 95% CI: 0.6—0.9), and GH (RR 0.46, 95% CI: 0.3—0.80) compared to those without epilepsy. A volcano plot (Fig. 2B) illustrates the magnitude and direction of these associations, with stratified data provided in Supplemental Table 3.

In *HEAL*, 28 protein biomarkers were measured (Supplemental Table 1). In addition to IL-1β, three proteins showed nominal associations with acquired epilepsy (IL-1RA, TNF-α, UCHL1) and one of the three remained significant after FDR adjustment. Neonates with acquired epilepsy had higher concentrations of IL-1β (RR 1.6, 95% CI 1.01—2.7) and UCHL1 (RR 3.5, 95% CI: 1.6—7.6) compared to those without epilepsy. Cross-cohort analysis identified IL-1β and UCHL1 as consistent biomarkers of acquired epilepsy (Fig. 2C). GH was not measured in *HEAL*, precluding validation; given its effect size in *NSR-RISE*, GH was considered exploratory in downstream model performance analyses.

Protein biomarker concentrations did not differ by infant sex in either cohort (Supplemental Table 4). In *NSR-RISE*, several biomarker concentrations varied by acute provoked seizure etiology (Supplemental Table 5), but these were not associated with acquired epilepsy.

### Prognostic model performance

**Fig. 3:**
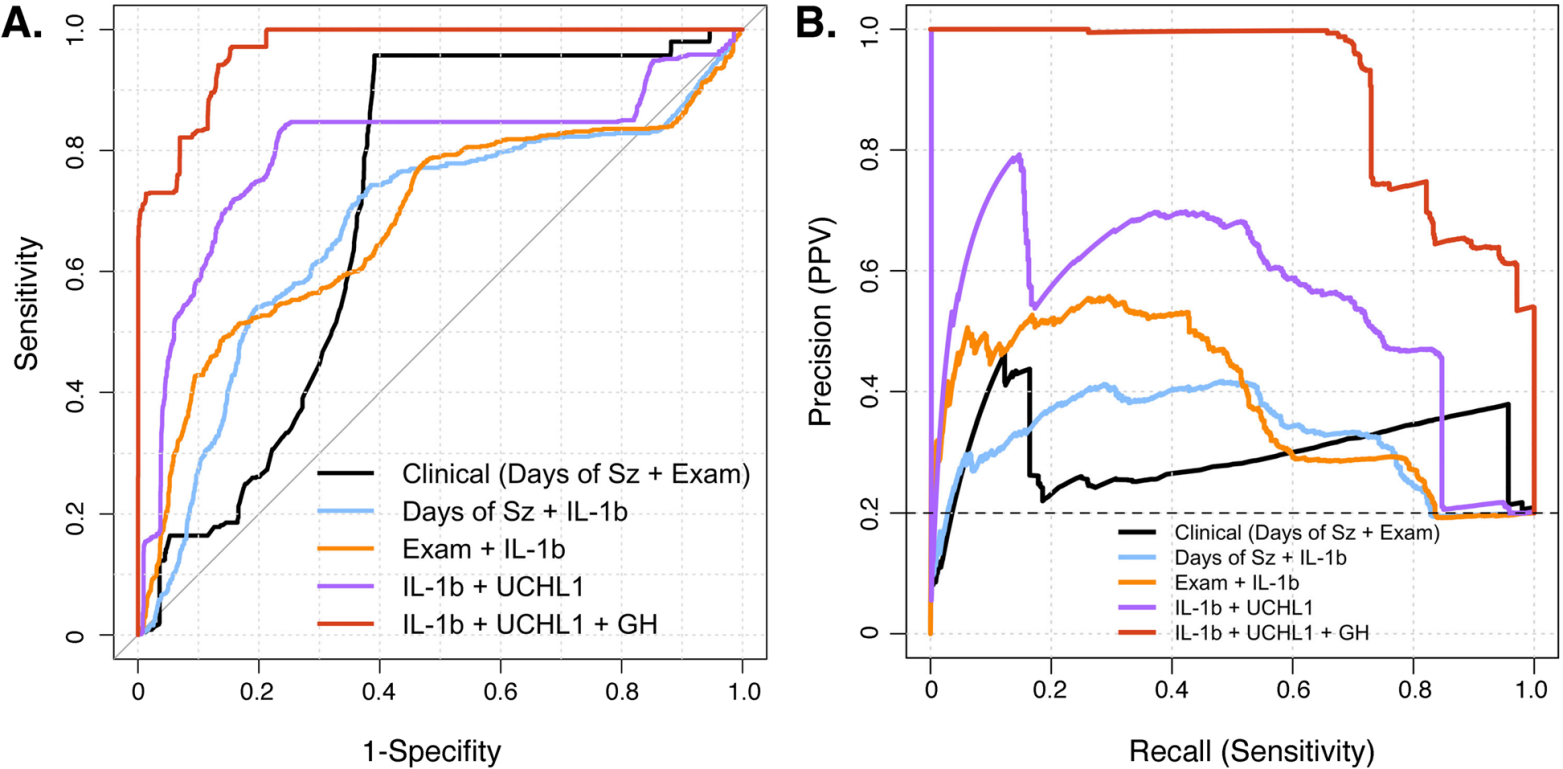
Addition of plasma biomarkers improves prediction of outcomes beyond clinical features in the *NSR-RISE* cohort. **A** Receiver operating characteristic (ROC) curves comparing model performance using clinical variables alone (≥ 3 days of EEG seizures, abbreviated “Days of Sz,” and abnormal neurological examination at discharge, abbreviated “Exam”) (black); “Days of Sz” + IL-1β (blue); “Exam” + IL-1β (orange); IL-1β + UCHL1 (purple); and IL-1β + UCHL1 + GH (red). Incorporation of biomarker data increased sensitivity and overall model discrimination compared with clinical features alone. **B** Precision–recall curves for the same models showing improved positive predictive value (PPV) and recall when biomarkers included

A clinical model including ≥ 3 days of EEG seizures and an abnormal neurological examination at discharge yielded an AUC of 0.71 (95% CI, 0.69—0.73; Fig. 3A). The addition of IL-1β to either ≥ 3 days of EEG seizures (AUC 0.67 (95% CI, 0.65—0.70) or abnormal neurological examination (AUC 0.68 (95% CI, 0.66—0.71) did not significantly improve model discrimination compared with the clinical model alone. In contrast, a biomarker-only model with IL-1β and UCHL1 increased the AUC to 0.80 (95% CI 0.78—0.82; *p* < 0.001), and further inclusion of GH achieved high discrimination (AUC 0.97; 95% CI: 0.96—0.97; *p* < 0.001).

We next evaluated model performance using precision-recall metrics. In *NSR-RISE*, acquired epilepsy occurred in 20% of participants, establishing a baseline precision of 0.20 (Fig. 3B). The clinical model achieved a mean AUPRC of 0.30 (95% CI, 0.28—0.32), modestly above baseline. The addition of IL-1β to ≥ 3 days of EEG seizures (AUPRC 0.32 (95% CI, 0.30—0.34; *p* = 0.08) did not improve performance, whereas addition to abnormal neurological examination produced a modest but significant gain (AUPRC 0.38 (95% CI, 0.35—0.42; *p* < 0.001). A biomarker-only model with IL-1β and UCHL1 further improved discrimination (AUPRC 0.55 (95% CI 0.51—0.58; *p* < 0.001), and inclusion of GH achieved high performance (AUPRC 0.91 (95% CI, 0.89—0.92; *p* < 0.001).

### Pathway analysis

**Fig. 4:**
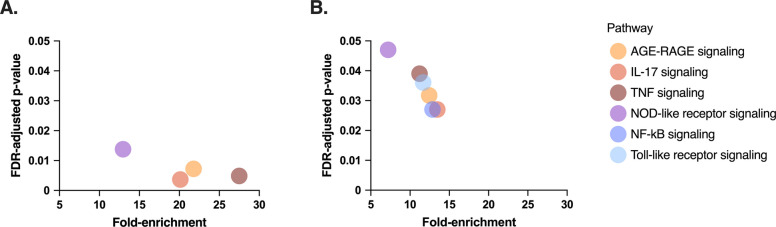
Pathway enrichment of protein biomarkers associated with acquired epilepsy after acute provoked neonatal seizures. Bubble plots show active subnetwork pathway enrichment results for the KEGG protein–protein interaction (PPI) network in the (**A**) *NSR-RISE* derivation cohort and the (**B**) *HEAL* validation cohort

To provide biological context for protein-level associations, pathway enrichment analyses were performed to identify convergent signaling networks linked to acquired epilepsy after acute provoked neonatal seizures. In *NSR-RISE*, epilepsy-associated proteins demonstrated enrichment of innate immune pathways, including TLR/IL-1/NF-κB cascades (AGE-RAGE, NOD-like receptor, and TNF signaling) and MAPK-associated IL-17 signaling (Fig. 4A). Validation analyses in *HEAL* demonstrated a similar enrichment profile, again highlighting AGE-RAGE, IL-17, NF-κB, TLR, and TNF pathways (Fig. 4B). Given the targeted nature of the inflammatory protein panel, these findings should be interpreted as exploratory and hypothesis-generating. Within this context, IL-1β mapped to the TLR/IL-1/NF-κB-related pathways, UCHL1 to MAPK and TNF-associated stress-response pathways consistent with neuronal injury, and GH to JAK/STAT and MAPK signaling modules associated with neurotrophic and reparative processes.

### miRNA biomarker analyses

**Fig. 5:**
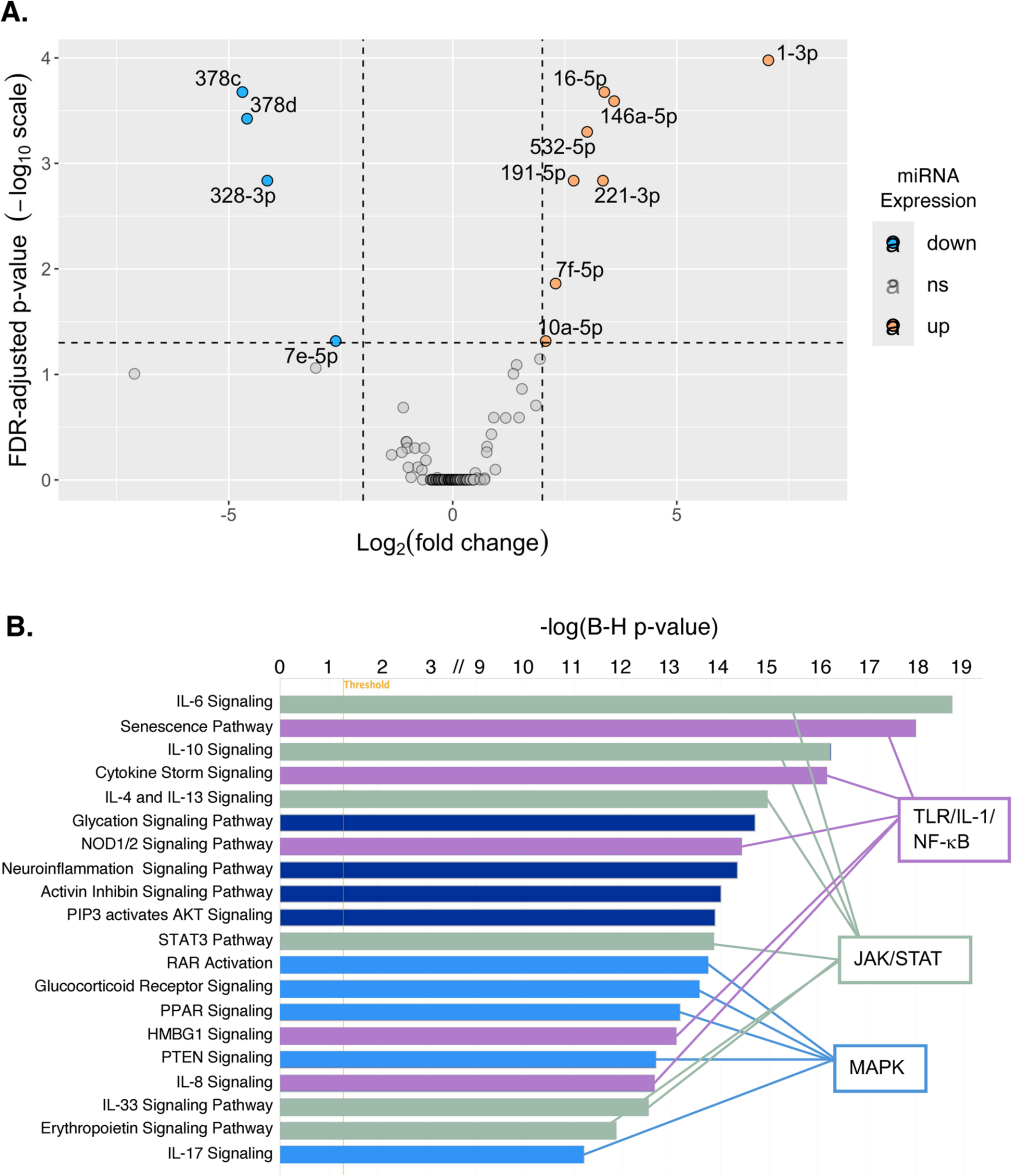
Differentially expressed microRNAs (miRNAs) and enriched inflammatory signaling pathways. **A** Volcano plot of circulating miRNAs showing significant differential expression between neonates with and without acquired epilepsy after acute provoked neonatal seizures. The horizontal dashed line indicates the false discovery rate (FDR) threshold (adjusted *p* < 0.05). The vertical dashed line indicates a ≥ fourfold difference in expression. **B** Pathway enrichment analysis of differentially expressed miRNAs identified enrichment of inflammatory and immune-related signaling cascades. The x-axis represents log10-transformed Benjamini-Hochberg (B-H) corrected *p*-value

miRNA sequencing was performed in a subset of 21 *NSR-RISE* participants, including all neonates who developed acquired epilepsy (*n* = 7) and a 2:1 etiology-matched group of neonates with acute provoked seizures who did not develop epilepsy (*n* = 14). All samples met QC thresholds for read depth and annotation. Length distribution analysis revealed reads were in the range of 20—30 nucleotides, consistent with miRNAs. Differential expression analysis identified 11 miRNAs associated with acquired epilepsy (FDR *p* < 0.05, ≥ fourfold change; Fig. 5A, Supplemental Table 6). Several of these, including miR-146a-5p and let-7f-5p, have been associated with TLR4/IL-1β-mediated NF-κB activation and downstream modulation of MAPK and JAK/STAT signaling [28, 63, 64]. Many of the differentially expressed miRNAs (miR-146a-5p, let-7e-5p, let-7f-5p, miR-328—3p, and miR-191—5p) are highly enriched in brain tissue, whereas others (miR-1—3p, miR-16—5p, miR-221—3p, miR-532—5p, and the miR-378 family) show broader systemic expression, consistent with both neural and peripheral immune sources [65].

Similar to protein pathway analyses, exploratory pathway analyses of differentially expressed miRNAs (Fig. 5B) identified enrichment of three super-pathway families: TLR/IL-1/NF-κB mediated innate immune activation (cytokine storm, NOD1/2, HMGB1, and IL-8 signaling), MAPK-associated cascades (IL-17, PTEN, PPAR, RAR, and glucocorticoid receptor signaling), and cytokine-driven JAK/STAT signaling (IL-6, IL-10, IL-4/IL-13, and IL-33, EPO, and STAT3 signaling).

### Weighted gene co-expression analysis (WGCNA)

**Fig. 6:**
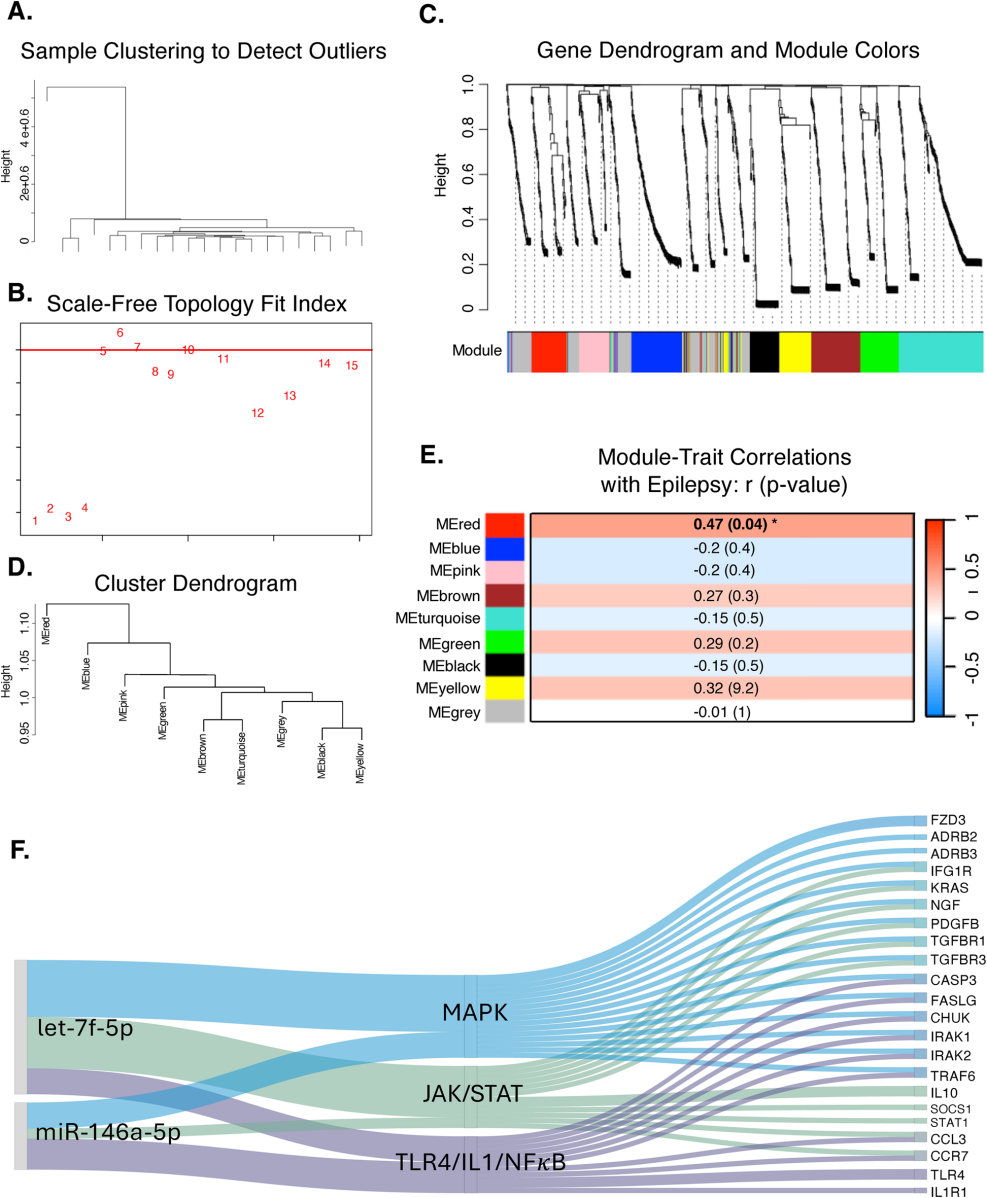
WGCNA links microRNAs with signaling pathways associated with acquired epilepsy after acute provoked neonatal seizures. **A**-**B** Weighted gene co-expression network analysis (WGCNA) quality control plots demonstrate sample clusters for outlier detection (**A**) and scale-free topology model fit (**B**). **C** miRNA dendrogram with original and merged module after dynamic tree cut and module merger. **D** Cluster dendrogram of module eigengenes visualize module relatedness. **E** Heatmap of module-trait correlations with acquired epilepsy after acute provoked neonatal seizures. **F** Sankey diagram mapping epilepsy-associated hub miRNAs (let-7f-5p, miR-146a-5p) to canonical signaling pathways and their downstream gene targets

Sample clustering identified one outlier (an infant with HIE without epilepsy), who was excluded to avoid biasing network topology (Fig. 6A). A soft-thresholding power of β = 6 was selected (Fig. 6B). These quality control steps ensured robust module detection in subsequent WGCNA analyses. Hierarchical clustering identified distinct co-expression modules that were subsequently merged by eigengene similarity (Fig. 6C-D). WGCNA identified one module (red) significantly correlated with acquired epilepsy (r = 0.47, *p* = 0.04, Fig. 6E). Candidate driver miRNAs associated with acquired epilepsy included let-7a-5p (MM: 0.95, GS *p*-value < 0.05), let-7f-5p (MM: 0.98, GS *p*-value < 0.05), miR-146a-5p (MM: 0.98, GS *P*-value < 0.05), and miR-200c-3p (MM: 0.91, GS *p*-value < 0.05). All are highly expressed in brain tissue. Notably, let-7f-5p and miR-146a-5p were also among the differentially expressed miRNAs. We conducted target mapping of these two species—let-7f-5p (170 mRNAs) and miR-146a (56 mRNAs)—because they were both hub driver miRNAs and significantly differentially expressed in neonates with acquired epilepsy. Pathway enrichment again implicated three super-pathway families: TLR/IL-1/NF-κB mediated innate immune activation, MAPK-associated cascades, and cytokine-driven JAK/STAT signaling (Fig. 6F; Supplemental Table 7).

## Discussion

In this prospective, multicenter analysis of neonates with acute provoked seizures, we identified reproducible plasma biomarkers that were associated with the development of acquired epilepsy. Our findings underscore the potential of blood-based biomarkers to provide objective, early prognostic information for neonates with acute provoked seizures. The strongest and most consistent associations were observed for IL-1β, a central pro-inflammatory cytokine, and UCHL1, a neuron-specific protein released after axonal injury. IL-1β and UCHL1 were both significantly elevated in neonates who later developed epilepsy across two independent populations—the *NSR-RISE* cohort (mixed seizure etiologies) and the *HEAL* cohort (seizures due to HIE). The incorporation of IL-1β and UCHL1 into prognostic models that included established clinical predictors of acquired epilepsy improved prognostic performance, while the addition of GH in an exploratory model further increased discrimination to high levels. A biomarker-only model with IL-1β, UCHL1, and GH demonstrated higher performance than models based on clinical features alone in this cohort. The superior performance of the biomarker-only model likely reflects the ability of IL-1β, UCHL1, and GH to capture mechanistically distinct yet complementary components of epileptogenesis, whereas clinical predictors represent downstream and variably expressed correlates of these biological processes. Biomarker-driven risk stratification may enable early identification of neonates at highest risk for epileptogenesis, thereby improving cohort enrichment and readiness for future clinical trials of anti-epileptogenic therapies.

In addition to plasma proteins, we found that miRNA expression profiles provided insight into biological pathways associated with epileptogenesis. Several brain-enriched miRNAs were differentially expressed in children who developed acquired epilepsy compared with those who did not, which supports the ability of peripherally collected circulating biomarkers to reflect CNS disease processes. Integrative pathway analyses were consistent with enrichment of TLR/IL-1/NF-κB, MAPK, and JAK/STAT cascades, which overlap with targets of current anti-inflammatory therapies with translatable potential [66–68]. Notably, miR-146a-5p and let-7f-5p emerged as hub regulators (“drivers”) of epilepsy-associated modules, supporting their biological relevance. However, unlike protein biomarkers such as IL-1β, UCHL1, and GH that demonstrate near-term translational potential, miRNA utility is constrained by reliance on sequencing-based assays and long turnaround times. At present, miRNAs are best positioned as exploratory mechanistic tools, but with advances in rapid detection technologies they could ultimately be integrated into multi-omic biomarker panels to refine risk prediction.

Taken together, these protein and miRNA findings suggest convergence on shared inflammatory pathways. Convergence of the protein data on innate immune pathways should be interpreted in the context of the targeted inflammatory biomarker panel, where enrichment of these pathways is expected. In contrast, miRNA profiling was performed using an unbiased approach, and demonstrated similar pathway enrichment, providing orthogonal support for these biological signals. Accordingly, our findings extend and validate prior observations that link inflammation and neuronal injury to epileptogenesis [7–9].

Within this framework, the association between IL-1β and acquired epilepsy warrants particular emphasis. IL-1β is a central effector of inflammasome activation, particularly through the NLRP3 complex, which promotes cleavage of pro–IL-1β into its active form and amplifies downstream NF-κB signaling [69]. Activation of this pathway increases neuronal excitability through modulation of NMDA receptor phosphorylation and reduction of GABAergic inhibition [11, 12]. In experimental animal models, pharmacologic inhibition of IL-1 signaling or upstream inflammasome pathways reduces seizure susceptibility and epileptogenesis, supporting the relevance of this pathway [15, 16]. Human data support these observations. Elevated IL-1β concentrations have been reported in plasma and cerebrospinal fluid in patients with febrile seizures and febrile status epilepticus, [70, 71] and genetic variation in IL-1 pathway genes has been associated with acquired epilepsy following brain injury [72, 73]. Together, these data support a model in which IL-1β reflects both activation of inflammasome-driven inflammatory cascades and a biologically relevant mechanism of epileptogenesis. Our findings extend this framework by demonstrating that circulating IL-1β measured during the subacute post-injury period is associated with later epilepsy risk in human neonates.

Complementing this inflammatory signal, UCHL1 represents a neuron-specific ubiquitin hydrolase released into circulation after axonal damage [74]. It has been detected in blood after neonatal brain injury and is a predictive biomarker of adverse outcome in adults with traumatic brain injury [47, 75]. UCHL1 may also modulate inflammatory pathways through MAPK and NF-κB [76]. Our results link plasma UCHL1 with epilepsy risk after neonatal brain injury, which supports the role of acute neuronal injury in long-term epileptogenesis.

Growth hormone (GH) represents a third biologically distinct signal. GH exerts trophic effects through JAK2/STAT(1/3/5) signaling, which supports neuronal survival, neurogenesis, synaptic plasticity, and myelination, and is considered a potential neuroprotective factor [77, 78]. GH deficiency has been linked to impaired recovery and cognitive deficits after brain injury, while GH supplementation may improve outcomes among adults with traumatic brain injury [79, 80]. The strong predictive value of GH in *NSR-RISE*, combined with its established trophic functions, suggests that reduced GH signaling may reflect a loss of neuroprotective JAK/STAT activity.

Taken together, these findings suggest that multiple biologically distinct processes contribute to epileptogenesis after neonatal brain injury. The etiologic heterogeneity of acute provoked neonatal seizures is an important consideration when interpreting these findings. Distinct injury mechanisms such as hypoxic-ischemic encephalopathy, stroke, and infection may differentially activate inflammatory pathways, both in magnitude and temporal profile. For example, infectious etiologies may be associated with more sustained inflammatory signaling, whereas hypoglycemia may reflect more transient metabolic and excitotoxic stress, although these patterns are not well defined in human neonates. In our cohort, no neonates with intracranial hemorrhage developed epilepsy, despite the recognized pro-inflammatory and epileptogenic effects of blood breakdown products [81]. Although this likely reflects the small sample size, it also raises the possibility that hemorrhage-related inflammatory responses differ from other injury types or that additional modifiers influence epileptogenic risk. Our analysis focused on shared downstream pathways associated with epileptogenesis, but upstream inflammatory responses may vary by etiology. Larger studies will be required to disentangle etiology-specific from convergent mechanisms.

At the regulatory level, our miRNA analyses similarly extend prior literature that links inflammation and neuronal injury to epileptogenesis. In animal models, miR-146a-5p is consistently upregulated after acute provoked seizures, and acts as a negative regulator of TLR/IL-1/NF-κB signaling [82–84]. Similar to peripheral IL-1 blockade, intracerebroventricular delivery of miR-146a mimics can modify epileptogenesis after brain injury in animal models [15]. However, non-invasive intranasal delivery may also modify ictagenesis [85]. In contrast, let-7f-5p, a member of the brain-enriched let-7 family, exerts dual effects where intracellularly it regulates neuronal differentiation, [86] but when released extracellularly it activates TLR7, and thereby amplifies neuroinflammation and excitotoxicity in rodent seizure models [63, 87]. Together, these findings provide biological context for the identification of miR-146a-5p and let-7f-5p as hub regulators (“drivers”) of epilepsy-associated modules in our cohort and reinforce their biological plausibility. Convergence of these miRNAs on IL-1/TLR/NF-κB and JAK/STAT signaling pathways further supports their value as mechanistic biomarkers that could ultimately be integrated with protein-based panels to refine risk prediction.

Several study limitations warrant consideration. First, sample sizes were modest and the number of children who developed acquired epilepsy over the course of follow-up was small, which limits statistical power. Given the small sample and the use of false discovery rate correction (FDR) to select candidate biomarkers for prognostic evaluation, logistic regression models incorporating UCHL1 and, subsequently, GH may be subject to optimism. The three-biomarker model (IL-1β, UCHL1, and GH) is further prone to overfitting, as all other models were constrained to two covariates to maintain model parsimony. However, inclusion of this exploratory model demonstrates the potential incremental prognostic value of combining biomarkers that represent complementary pathophysiological processes—namely inflammation, neuronal injury, and impaired trophic signaling. Second, although the association of IL-1β and UCHL1 with acquired epilepsy was replicated across cohorts, GH was not measured in the *HEAL* cohort. Third, plasma concentrations of a few proteins (e.g. UCHL1) approached assay detection thresholds, which could underestimate effect sizes.

Additional limitations relate to clinical characterization and cohort design. Seizure burden was characterized using duration-based metrics; more granular measures such as cumulative seizure burden may improve future models. We also did not include a comparator cohort of infants with similar brain injury but without neonatal seizures. Neonates with acute provoked seizures represent a high-risk group in whom epilepsy typically develops within the first two years of life, whereas risk is lower and more delayed in infants without seizures [35, 88, 89]. Finally, while biomarker associations support biological plausibility, causality cannot be inferred; IL-1β and UCHL1 may represent mediators of epileptogenesis, markers of injury severity, or both. Genetic susceptibility may contribute acquired epilepsy risk, and this remains an important area for future investigation, and is a central objective of the *NSR-GENE* study (*Neonatal Seizure Registry, GEnetics of Post-Neonatal Epilepsy*—NCT05361070).

## Conclusions

In summary, these findings identify IL-1β, UCHL1, and GH as a core set of plasma biomarkers associated with acquired epilepsy after acute provoked neonatal seizures. Circulating miRNAs—particularly miR-146a-5p and let-7f-5p—may provide mechanistic insights into dysregulated inflammatory signaling, reinforcing the hypothesis that inflammation secondary to neuronal injury contributes to long-term risk of acquired epilepsy. Future work should incorporate longitudinal biomarker profiling to define temporal dynamics, replicate our GH findings, and establish clinically meaningful biomarker thresholds alongside implementation strategies for bedside biomarker measurements to establish clinical trial readiness [75, 90, 91]. Additional preclinical studies that pharmacologically target the IL-1 axis and JAK/STAT signaling pathways in models of epileptogenesis are needed to elucidate causal mechanisms and inform therapeutic strategies [92–94]. Ultimately, the integration of these biomarkers into prospective clinical trials could enable precision prevention of epilepsy in high-risk neonates.

## Supplementary Information

Supplementary Material 1. Supplemental Table 1: Proteins measured in the NSR-RISE and HEAL cohorts. Supplemental Table 2: HEAL cohort with acute provoked seizures and biomarker data, stratified by whether participants had follow-up data for acquired epilepsy. Supplemental Table 3: Protein biomarker concentrations in the NSR-RISE cohort by epilepsy status at 2-years of age. Supplemental Table 4: Protein biomarker concentrations in the NSR-RISE cohort by sex. Supplemental Table 5: Protein biomarker concentrations in the NSR-RISE cohort by acute provoked seizure etiology. Supplemental Table 6: Statistically significant differentially expressed microRNAs (miRNA) between children with and without acquired epilepsy after neonatal acute provoked seizures in the NSR-RISE cohort. Supplemental Table 7: microRNA (miRNA) to messenger (mRNA) targets for hub genes identified on Weighted gene co-expression network analysis (WGCNA).

## Data Availability

The minimal dataset necessary to interpret and replicate the findings is not publicly available due to protection of participant privacy and institutional data-sharing policies involving human subjects research. De-identified datasets generated or analyzed during the current study are available from the corresponding author upon reasonable request and subject to institutional approvals and data use agreements. Analytic code supporting the findings of this study is publicly available at: https://github.com/aln142/NSR-RISE.git

## Abbreviations

ASM: Anti-seizure medication
AUC: Area under the (receiver operating characteristic) curve
AUPRC: Area under the precision–recall curve
BDNF: Brain-derived neurotrophic factor
BSID-III: Bayley Scales of Infant and Toddler Development, Third Edition
C5a: Complement component 5a
CCL: Chemokine (C–C motif) ligand
cEEG: (Continuous video)-electroencephalography
CNS: Central nervous system
CX3CL1: Fractalkine (C–X3–C motif chemokine ligand 1)
EDTA: Ethylenediaminetetraacetic acid
EPO: Erythropoietin
FDR: False discovery rate
GFAP: Glial fibrillary acidic protein
GH: Growth hormone
GMFCS: Gross Motor Function Classification System
HEAL: High-dose Erythropoietin for Asphyxia and EncephaLopathy
HIE: Hypoxic-ischemic encephalopathy
HMGB1: High mobility group box 1
ICAM-1: Intercellular adhesion molecule 1
IFN-γ: Interferon gamma
IL: Interleukin
IL-1RA: Interleukin-1 receptor antagonist
IPA: Ingenuity Pathway Analysis
IQR: Interquartile range
JAK/STAT: Janus kinase / signal transducer and activator of transcription
KEGG: Kyoto Encyclopedia of Genes and Genomes
MAPK: Mitogen-activated protein kinase
MCP-1: Monocyte chemoattractant protein 1
miRNA: MicroRNA
MIP-1α: Macrophage inflammatory protein 1 alpha
MM: Module membership
MRI: Magnetic resonance imaging
NCAM-1: Neural cell adhesion molecule 1
NF-κB: Nuclear factor kappa B
NGB: Neuregulin-1-β-1
NOD: Nucleotide-binding oligomerization domain
NSE: Neuron-specific enolase
NSR: Neonatal Seizure Registry
NSR-RISE: Neonatal Seizure Registry – Role of Inflammation after Neonatal Seizures and Later Development of Epilepsy
PPI: Protein–protein interaction
PPV: Positive predictive value
ROC: Receiver operating characteristic
RPM: Reads per million
RR: Relative risk
SD: Standard deviation
s100b: S100 calcium-binding protein B
TLR: Toll-like receptor
TNF-α: Tumor necrosis factor alpha
TOM: Topological overlap matrix
UCHL1: Ubiquitin carboxy-terminal hydrolase L1
VCAM-1: Vascular cell adhesion molecule 1
VEGF: Vascular endothelial growth factor
WGCNA: Weighted gene co-expression network analysis
WIDEA-FS: Warner Initial Developmental Evaluation of Adaptive and Functional Skills
