# Supplementary material for: Mechanistically informed circulating biomarkers are associated with acquired epilepsy after neonatal brain injury
Source: J Neuroinflammation. 2026 May 9;23:215. doi: 10.1186/s12974-026-03853-9 (PMC13312635; doi:10.1186/s12974-026-03853-9)
Supplement: Supplementary file 1 — Supplementary Material 1. Supplemental Table 1: Proteins measured in the NSR-RISE and HEAL cohorts. Supplemental Table 2: HEAL cohort with acute provoked seizures and biomarker data, stratified by whether participants had follow-up data for acquired epilepsy. Supplemental Table 3: Protein biomarker concentrations in the NSR-RISE cohort by epilepsy status at 2-years of age. Supplemental Table 4: Protein biomarker concentrations in the NSR-RISE cohort by sex. Supplemental Table 5: Protein biomarker concentrations in the NSR-RISE cohort by acute provoked seizure etiology. Supplemental Table 6: Statistically significant differentially expressed microRNAs (miRNA) between children with and without acquired epilepsy after neonatal acute provoked seizures in the NSR-RISE cohort. Supplemental Table 7: microRNA (miRNA) to messenger (mRNA) targets for hub genes identified on Weighted gene co-expression network analysis (WGCNA). [file 12974_2026_3853_MOESM1_ESM.docx]

**Supplemental Files:**

Supplemental Table 1: Proteins measured in the *NSR-RISE* and *HEAL* cohorts.

Supplemental Table 2: *HEAL* cohort with acute provoked seizures and biomarker data, stratified by whether participants had follow-up data for acquired epilepsy.

Supplemental Table 3: Protein biomarker concentrations in the *NSR-RISE* cohort by epilepsy status at 2-years of age

Supplemental Table 4: Protein biomarker concentrations in the *NSR-RISE* cohort by sex

Supplemental Table 5: Protein biomarker concentrations in the *NSR-RISE* cohort by acute provoked seizure etiology

Supplemental Table 6: Statistically significant differentially expressed microRNAs (miRNA) between children with and without acquired epilepsy after neonatal acute provoked seizures in the *NSR-RISE* cohort.

Supplemental Table 7: microRNA (miRNA) to messenger (mRNA) targets for hub genes identified on Weighted gene co-expression network analysis (WGCNA).

**Supplemental Table 1: Proteins measured in the *NSR-RISE* and *HEAL* cohorts.**

| Protein | Protein Symbol | Measured in *NSR-RISE* | Measured in *HEAL* |
| --- | --- | --- | --- |
| Brain-derived neurotrophic factor | BDNF | Yes | Yes |
| Complement component 5a | C5a | Yes | Yes |
| CX3CL1/Fractalkine | CX3CL1 | Yes | Yes |
| Erythropoietin | EPO | No | Yes |
| Glial fibrillary acidic protein | GFAP | Yes | Yes |
| Growth Hormone | GH | Yes | No |
| Intercellular adhesion molecule 1 / Cluster of differentiation (CD)54 | ICAM-1 | Yes | Yes |
| Interferon-γ | IFN-γ | Yes | Yes |
| Interleukin (IL)-1β | IL-1β | Yes | Yes |
| IL-10 | IL-10 | Yes | Yes |
| IL-12p70 | IL-12p70 | Yes | Yes |
| IL-13 | IL-13 | Yes | Yes |
| IL-17A | IL-17A | Yes | Yes |
| IL-1 receptor antagonist | IL-1RA | Yes | Yes |
| IL-21 | IL-21 | Yes | No |
| IL-23 | IL-23 | Yes | No |
| IL-33 | IL-33 | Yes | Yes |
| IL-4 | IL-4 | Yes | No |
| IL-5 | IL-5 | Yes | No |
| IL-6 | IL-6 | Yes | Yes |
| IL-8 | IL-8 | Yes | Yes |
| Monocyte chemoattractant protein 1 -1/ Chemokine ligand (CCL)2 | MCP-1/CCL2 | Yes | Yes |
| Macrophage inflammatory protein 1α/CCL3 | MIP1α/CCL3 | Yes | Yes |
| Macrophage inflammatory protein 1β/CCL4 | MIP-1β/CCL4 | Yes | Yes |
| Neural cell adhesion molecule 1/CD56 | NCAM-1 | Yes | Yes |
| Neuroregulin-1-b-1 | NGB | Yes | Yes |
| Neuron-specific enolase | NSE | Yes | Yes |
| Tumor necrosis factor-α | TNF-α | Yes | Yes |
| Tau | Tau | Yes | Yes |
| Ubiquitin carboxy-terminal hydrolase-L1 | UCHL1 | Yes | Yes |
| Vascular cell adhesion molecule 1/CD106 | VCAM1 | Yes | Yes |
| Vascular endothelial growth factor | VEGF | Yes | Yes |
| S100 calcium-binding protein B | s100b | Yes | Yes |

**Supplemental Table 2: *HEAL* cohort with acute provoked seizures and biomarker data, stratified by whether participants had follow-up data for acquired epilepsy.**

| **Characteristics** | **Overall** | **Yes** | **No** | **p-value** |
| --- | --- | --- | --- | --- |
|  | N=71 | N=40 | N=31 |  |
| Maternal characteristics | | | | |
| Maternal race (%) | | | | 0.54 |
| White | 53 (75) | 31 (78) | 22 (71) |  |
| Black | 10 (14) | 5 (13) | 5 (16) |  |
| Asian | 2 (3) | 0 | 2 (7) |  |
| Multiple/other/unknown | 6 (9) | 4 (10) | 2 (7) |  |
| Hispanic ethnicity, n (%) | 16 (23) | 9 (23) | 7 (23) | 1.0 |
| Education, ≤ high school, n(%) | 30 (42) | 15 (38) | 15 (48) | 0.50 |
| Outborn delivery, n (%) | 66 (93) | 38 (95) | 28 (90) | 0.77 |
| Maternal chorioamnionitis, n (%) | 8 (11) | 4 (10) | 4 (13) | 1.0 |
| Cesarean section delivery = Yes (%) | 55 (78) | 33 (83) | 22 (71) | 0.39 |
| Any sentinel event, n (%)^a^ | 27 (38) | 15 (38) | 12 (39) | 1.0 |
| Neonatal Characteristics | | | | |
| Gestational age, wk, mean (SD) | 39.1 (1.5) | 38.9 (1.4) | 39.5 (1.7) | 0.11 |
| Birth weight, kg, mean (SD) | 3.3 (0.7) | 3.3 (0.6) | 3.3 (0.8) | 0.57 |
| Female, n (%) | 33 (47) | 18 (45) | 15 (48) | 0.965 |
| 5-minute Apgar, median [IQR] | 2 [1,3] | 2 [1,4] | 3 [1,3] | 0.43 |
| 10-minute Apgar, median [IQR] | 4 [2,5] | 4 [3,5] | 3 [2,5] | 0.37 |
| Continued resuscitation at 10 min, n (%)^b^ | 67 (94) | 37 (93) | 30 (97) | 0.80 |
| Lowest pH, mean (SD)^c^ | 6.9 (0.2) | 6.9 (0.2) | 6.9 (0.2) | 0.30 |
| Minimum base deficit, mean (SD)^c^ | -20 (7) | -20 (7) | -21 (7) | 0.48 |
| **Severe encephalopathy, n (%)** | **34 (49)** | **14 (35)** | **20 (65)** | **0.03** |
| Erythropoietin treatment arm, n (%) | 32 (45) | 17 (43) | 15 (48) | 0.80 |
| Neonatal Clinical Course | | | | |
| All oral feeding at discharge, n (%) | 38 (70) | 24 (62) | 14 (93) | 0.05 |
| 2-year Outcomes | | | | |
|  | n=54 | n=40 | n=14 |  |
| **CP (GMCSF ≥ 1), n (%)^c^** | **13 (18)** | **13 (32)** | **0** | **0.001** |
| **Total WIDEA, mean (SD)** | **150 (42)** | **139 (45)** | **177 (14)** | **0.03** |
| **Receiving Services, n (%)^e^** | **31 (57)** | **27 (68)** | **4 (29)** | **0.03** |

Abbreviation: CP: Cerebral palsy; GMCSF: Gross motor function classification system; IQR: Interquartile range; WIDEA: Warner Initial Developmental Evaluation of Adaptive and Functional Skills – Full Scale.

*p < 0.05, ** p < 0.001, *** p <0.001. All other p-values are ns (not significant).

^a^ A sentinel event was defined as shoulder dystocia, placental abruption, prolapsed cord, or uterine rupture.

^b^ Ongoing resuscitation with chest compressions, mechanical ventilation, or both was warranted at 10 minutes of age.

^c^ Shown is the lowest pH or worst base deficit among cord arterial, cord venous, and arterial blood gas samples obtained tained before 60 minutes of age.

^d^ Determined by chart review.

^f^ Including physical therapy, occupational therapy, speech and language therapy, vision therapy, and/or early intervention.

**Supplemental Table 3: Protein biomarker concentrations in the *NSR-RISE* cohort by epilepsy status at 2-years of age.** Biomarker concentrations in pg/mL. Significant differences between biomarker concentrations are detailed in the results section of the manuscript and in Figure 2A.

| Protein Biomarker | Epilepsy | No Epilepsy |
| --- | --- | --- |
|  | N=7 | N=28 |
| IL-1RA (median [IQR]) | 7276.00 [3407.15, 15773.47] | 1560.49 [1139.36, 3673.53] |
| IL-10 (median [IQR]) | 8.37 [4.59, 11.16] | 6.05 [2.13, 16.12] |
| IL-13 (median [IQR]) | 2.79 [0.12, 4.90] | 0.12 [0.12, 9.74] |
| IL-33 (median [IQR]) | 3.13 [3.06, 3.52] | 3.13 [1.87, 4.64] |
| C5a (median [IQR]) | 29906.85 [21721.25, 53979.82] | 26944.50 [14068.64, 37332.16] |
| CX3CL1/Fractalkine (median [IQR]) | 127.02 [65.34, 142.22] | 54.93 [30.52, 91.81] |
| CD54/ICAM-1 (median [IQR]) | 632976.45 [627048.64, 692550.86] | 1079600.00 [542160.80, 1235875.00] |
| IFN-g (median [IQR]) | 6.43 [0.30, 11.67] | 0.94 [0.30, 7.24] |
| IL-1b (median [IQR]) | 3.88 [1.16, 4.84] | 0.25 [0.25, 1.84] |
| IL-4 (median [IQR]) | 9.18 [4.30, 38.98] | 3.25 [0.90, 68.66] |
| IL-5 (median [IQR]) | 2.86 [0.47, 4.16] | 2.92 [1.05, 6.11] |
| IL-6 (median [IQR]) | 5.32 [4.24, 43.53] | 5.39 [3.24, 19.57] |
| IL-8 (median [IQR]) | 11.32 [6.97, 12.61] | 21.62 [11.70, 48.78] |
| IL-12p70 (median [IQR]) | 0.92 [0.25, 2.28] | 0.25 [0.25, 0.80] |
| IL-17 (median [IQR]) | 5.58 [0.37, 23.95] | 0.37 [0.37, 1.70] |
| IL-21 (median [IQR]) | 0.33 [0.12, 1.29] | 0.12 [0.12, 0.48] |
| IL-23 (median [IQR]) | 78.68 [3.97, 242.60] | 15.07 [3.97, 130.26] |
| CCL2/MCP1 (median [IQR]) | 218.98 [199.93, 288.78] | 407.62 [222.64, 929.93] |
| CCL3/MIP-1a (median [IQR]) | 23.13 [19.87, 32.29] | 19.88 [15.04, 30.50] |
| CCL4/MIP-1b (median [IQR]) | 31.60 [28.13, 119.50] | 68.09 [46.76, 104.42] |
| CD106/VCAM-1 (median [IQR]) | 1869200.00 [1577850.00, 2081550.00] | 2908100.00 [2114375.00, 3972575.00] |
| TNF-a (median [IQR]) | 12.25 [11.13, 16.82] | 20.24 [14.67, 26.05] |
| GFAP (median [IQR]) | 2265.87 [1721.66, 9458.55] | 1967.48 [1132.14, 3070.49] |
| s100b (median [IQR]) | 662.61 [551.82, 1188.86] | 685.47 [373.53, 1343.09] |
| UCHL1 (median [IQR]) | 640.57 [600.79, 838.58] | 516.04 [514.43, 618.87] |
| TAU (median [IQR]) | 13616696.73 [8719708.68, 24814677.32] | 3465460.56 [2599816.87, 11493453.29] |
| BDNF (median [IQR]) | 149.02 [94.85, 234.14] | 200.89 [44.87, 854.96] |
| CD56/NCAM1 (median [IQR]) | 325030.48 [272318.36, 343593.96] | 306679.77 [280103.85, 349153.66] |
| GH (median [IQR]) | 3433.82 [2643.48, 8817.46] | 11808.22 [7920.73, 17300.42] |
| NGB (median [IQR]) | 11.18 [8.12, 12.81] | 11.18 [11.18, 11.18] |
| NSE (median [IQR]) | 7154.70 [5624.62, 11512.32] | 11376.77 [8354.20, 16358.74] |
| VEGF (median [IQR]) | 34.52 [31.12, 38.31] | 29.33 [20.27, 62.33] |

Abbreviations: IQR: Inter-quartile range. Biomarker names are listed in methods.

**Supplemental Table 4: Protein biomarker concentrations in the *NSR-RISE* cohort by sex.** Biomarker concentrations in pg/mL. No significant differences by sex were found.

| Protein Biomarker | Female | Male |
| --- | --- | --- |
|  | N=11 | N=24 |
| IL-1RA (median [IQR]) | 1876.95 [1295.31, 5080.98] | 1837.80 [1095.60, 4138.50] |
| IL-10 (median [IQR]) | 6.05 [2.13, 11.69] | 7.21 [2.71, 17.72] |
| IL-13 (median [IQR]) | 0.12 [0.12, 4.48] | 0.12 [0.12, 9.90] |
| IL-33 (median [IQR]) | 3.13 [3.06, 4.15] | 3.13 [1.97, 4.83] |
| C5a (median [IQR]) | 23707.82 [16350.18, 41257.83] | 28091.71 [14068.64, 37332.16] |
| CX3CL1/Fractalkine (median [IQR]) | 30.85 [28.48, 80.68] | 66.75 [45.44, 131.69] |
| CD54/ICAM-1 (median [IQR]) | 709420.15 [537993.28, 1136900.00] | 888638.94 [619339.66, 1225200.00] |
| IFN-g (median [IQR]) | 0.30 [0.30, 6.69] | 1.08 [0.30, 9.64] |
| IL-1b (median [IQR]) | 0.25 [0.25, 0.84] | 1.04 [0.25, 3.76] |
| IL-4 (median [IQR]) | 4.38 [0.90, 19.83] | 6.29 [0.90, 71.18] |
| IL-5 (median [IQR]) | 2.36 [0.87, 2.92] | 3.44 [1.05, 7.31] |
| IL-6 (median [IQR]) | 4.97 [3.62, 21.63] | 5.49 [2.99, 25.44] |
| IL-8 (median [IQR]) | 14.23 [11.53, 21.62] | 21.81 [9.53, 52.69] |
| IL-12p70 (median [IQR]) | 0.25 [0.25, 0.81] | 0.41 [0.25, 1.09] |
| IL-17 (median [IQR]) | 0.37 [0.37, 1.98] | 0.37 [0.37, 7.59] |
| IL-21 (median [IQR]) | 0.12 [0.12, 0.12] | 0.12 [0.12, 0.86] |
| IL-23 (median [IQR]) | 3.97 [3.97, 42.27] | 35.89 [3.97, 244.70] |
| CCL2/MCP1 (median [IQR]) | 269.98 [197.74, 637.38] | 357.14 [214.26, 1175.72] |
| CCL3/MIP-1a (median [IQR]) | 17.14 [10.43, 32.03] | 22.40 [17.36, 30.58] |
| CCL4/MIP-1b (median [IQR]) | 71.71 [34.06, 91.72] | 64.34 [33.72, 111.04] |
| CD106/VCAM-1 (median [IQR]) | 3178800.00 [2003850.00, 5156850.00] | 2422450.00 [1815025.00, 3272225.00] |
| TNF-a (median [IQR]) | 18.41 [12.17, 21.93] | 18.80 [12.26, 26.05] |
| GFAP (median [IQR]) | 2265.87 [1256.28, 5045.99] | 2031.32 [1132.14, 2942.34] |
| s100b (median [IQR]) | 662.61 [349.38, 1374.77] | 646.97 [403.38, 1343.09] |
| UCHL1 (median [IQR]) | 575.48 [526.88, 604.40] | 535.73 [514.43, 741.90] |
| TAU (median [IQR]) | 3548686.04 [2750639.76, 16194512.58] | 7306891.02 [2744710.00, 16710595.61] |
| BDNF (median [IQR]) | 242.58 [79.19, 955.08] | 154.66 [61.43, 279.86] |
| CD56/NCAM1 (median [IQR]) | 340408.62 [304927.63, 355382.06] | 299532.56 [273754.22, 330715.45] |
| GH (median [IQR]) | 10876.24 [7929.42, 16845.32] | 9911.08 [5510.87, 15820.24] |
| NGB (median [IQR]) | 11.18 [9.88, 11.18] | 11.18 [11.18, 11.18] |
| NSE (median [IQR]) | 10677.23 [8656.35, 16698.63] | 11291.12 [7357.94, 14941.40] |
| VEGF (median [IQR]) | 34.52 [26.82, 63.62] | 29.33 [17.46, 42.59] |

Abbreviations: IQR: Inter-quartile range. Biomarker names are listed in methods.

**Supplemental Table 5: Protein biomarker concentrations in the *NSR-RISE* cohort by acute provoked seizure etiology.** Biomarker concentrations in pg/mL.

| Protein Biomarker | HIE | Ischemic Stroke | Intracerebral Hemorrhage | Meningoencephalitis | **P-value**  **(4 group)** | **P-value**  **(3 group)^a^** |
| --- | --- | --- | --- | --- | --- | --- |
|  | N=18 | N=7 | N=5 | N=3 |  |  |
| IL-1RA (median [IQR]) | 3,265 [1,323, 5,235] | 1,302.83 [806.1, 1,655] | 1,623.72 [1,288, 4,321] | 1,029 [711.9, 1,604] | ns | ns |
| IL-10 (median [IQR]) | 14.23 [3.38, 29.66] | 4.66 [2.17, 11.69] | 3.13 [2.66, 3.20] | 8.37 [7.21, 8.95] | ns | ns |
| **IL-13 (median [IQR])** | **4.90 [0.12, 22.80]** | **0.12 [0.12, 0.12]** | **0.12 [0.12, 1.16]** | **1.24 [0.68, 2.02]** | 0.09 | ***** |
| IL-33 (median [IQR]) | 3.25 [2.02, 6.86] | 3.13 [1.94, 3.13] | 3.88 [3.06, 4.64] | 3.88 [3.47, 4.26] | ns | ns |
| C5a (median [IQR]) | 26908.48  [12964.68, 30186.30] | 40,225.98  [25239.39, 41921.76] | 23,707.82  [20859.72, 41229.14] | 26,753.24  [23151.08, 32422.22] | ns | ns |
| **CX3CL1/Fractalkine (median [IQR])** | **68.14 [52.81, 121.46]** | **45.68 [26.94, 61.59]** | **29.51 [27.45, 38.93]** | **141.90 [139.88, 143.34]** | ***** | ***** |
| CD54/ICAM-1 (median [IQR]) | 697,315.92  [597,534, 1,126,825] | 1,049,900.00  [583,013, 1,142,850] | 1,183,200  [5,338,16, 1,321,100] | 1,227,300  [1,012,236, 1,244,450] | ns | ns |
| **IFN-g (median [IQR])** | **5.78 [0.88, 10.49]** | **0.30 [0.30, 1.44]** | **0.30 [0.30, 0.30]** | **11.10 [5.87, 12.14]** | ***** | ***** |
| IL-1b (median [IQR]) | 0.56 [0.25, 3.27] | 0.25 [0.25, 0.70] | 0.25 [0.25, 0.25] | 3.74 [2.68, 4.50] | 0.07 | ns |
| IL-4 (median [IQR]) | 36.82 [0.90, 193.57] | 0.90 [0.90, 17.40] | 0.90 [0.90, 4.38] | 9.18 [7.03, 11.27] | ns | ns |
| IL-5 (median [IQR]) | 5.42 [1.23, 8.74] | 2.16 [1.50, 2.79] | 2.52 [0.94, 2.94] | 3.05 [2.96, 3.46] | ns | ns |
| IL-6 (median [IQR]) | 13.68 [5.30, 31.01] | 3.07 [1.57, 6.70] | 4.97 [4.67, 12.15] | 5.13 [3.78, 51.29] | ns | ns |
| **IL-8 (median [IQR])** | **42.25 [14.08, 58.08]** | **9.67 [9.15, 15.20]** | **19.75 [14.23, 23.49]** | **4.86 [4.58, 17.77]** | ***** | ***** |
| IL-12p70 (median [IQR]) | 0.60 [0.25, 0.92] | 0.25 [0.25, 0.25] | 0.25 [0.25, 0.85] | 2.14 [1.39, 2.43] | ns | ns |
| IL-17 (median [IQR]) | 0.37 [0.37, 4.82] | 0.37 [0.37, 0.89] | 0.37 [0.37, 0.37] | 17.93 [11.89, 21.98] | 0.08 | ns |
| **IL-21 (median [IQR])** | **0.12 [0.12, 0.51]** | **0.12 [0.12, 0.12]** | **0.12 [0.12, 0.12]** | **1.33 [1.03, 1.43]** | ***** | ns |
| IL-23 (median [IQR]) | 38.48 [3.97, 216.74] | 13.81 [3.97, 39.68] | 3.97 [3.97, 3.97] | 255.67 [190.70, 280.98] | ns | ns |
| CCL2/MCP1 (median [IQR]) | 462.36 [310.02, 2109.58] | 223.96 [184.26, 363.19] | 176.49 [171.37, 693.76] | 218.68 [209.85, 525.26] | ns | 0.09 |
| **CCL3/MIP-1a (median [IQR])** | **25.55 [19.28, 34.32]** | **15.51 [11.50, 16.50]** | **29.71 [8.42, 31.15]** | **18.77 [18.20, 19.17]** | 0.05 | ***** |
| CCL4/MIP-1b (median [IQR]) | 65.48 [38.18, 112.06] | 64.21 [39.72, 78.49] | 78.08 [38.71, 150.21] | 23.37 [21.85, 43.92] | ns | ns |
| CD106/VCAM-1 (median [IQR]) | 2,908,100  [1,987,375, 3,783,200] | 2,133,400.00  [1,678,750, 2,559,450] | 3,219,100  [3,051,600, 5,135,100] | 1,869,200  [1,760,850, 1,963,250] | 0.07 | ns |
| TNF-a (median [IQR]) | 20.19 [14.54, 33.20] | 12.20 [8.40, 18.84] | 21.22 [18.41, 26.66] | 17.34 [14.78, 19.09] | ns | ns |
| **GFAP (median [IQR])** | **2,456**  **[1345, 4,967]** | **1,732**  **[1,256, 3,248]** | **3,186**  **[2,569, 5,554]** | **785.3**  **[560.6, 900.0]** | ***** | ns |
| s100b (median [IQR]) | 617.4 [425.2, 2,271] | 401.3 [198.4, 605] | 1,094.66 [304.2, 1,332] | 1,332 [908.8, 1,494] | ns | ns |
| UCHL1 (median [IQR]) | 561.02 [514.43, 741.90] | 514.43 [514.43, 597.18] | 539.34 [514.43, 655.04] | 514.43 [514.43, 577.50] | ns | ns |
| **TAU (median [IQR])** | **9,219,032**  **[4,553,718, 2,986,2785]** | **2,343,794**  **[2,090,652, 3,763,435]** | **3,548,686**  **[3,296,753, 13,73,5732]** | **2,787,506**  **[2,209,994, 2,994,146]** | ***** | ***** |
| BDNF (median [IQR]) | 79.84 [47.32, 269.66] | 215.74 [93.08, 709.40] | 242.58 [112.88, 1415.75] | 248.37 [186.72, 863.48] | ns | ns |
| CD56/NCAM1 (median [IQR]) | 316,818  [271,959, 349,597] | 304,754  [257,465, 317,173] | 309,880  [299,975, 348,221] | 304,451  [299,533, 325,615] | ns | ns |
| GH (median [IQR]) | 9,911  [5,952, 17,531] | 10,876  [3,223, 15,955] | 14,219  [8,655, 15,279] | 7,556  [4,973, 12,312] | ns | ns |
| NGB (median [IQR]) | 11.18 [10.20, 11.18] | 11.18 [11.18, 11.18] | 11.18 [9.88, 11.18] | 11.18 [11.18, 11.18] | ns | ns |
| NSE (median [IQR]) | 11,192  [7,726, 15,933] | 11,304  [8,380, 13,757] | 15,396  [8492, 16,329] | 9,028  [7009, 12,329] | ns | ns |
| VEGF (median [IQR]) | 28.36 [19.56, 37.42] | 28.71 [8.69, 31.80] | 66.19 [61.05, 102.37] | 38.73 [33.72, 90.40] | 0.06 | 0.05 |

Abbreviations: IQR: Inter-quartile range. Biomarker names are listed in methods.

*P < 0.05, ** p < 0.001, *** p <0.00, Non-significant associations with p-values >0.05 and <0.01 are listed. All other p-values are ns (not significant).

^a^ 3-group comparison included Hypoxic-ischemic encephalopathy, ischemic stroke, and hemorrhagic stroke only given the low number of participants with meningoencphalitis.

**Supplemental Table 6: Statistically significant differentially expressed microRNAs (miRNA) between children with and without acquired epilepsy after neonatal acute provoked seizures in the *NSR-RISE* cohort.**

| miRNA ID : mature miRNA accession number | Fold Change | FDR P-value |
| --- | --- | --- |
| hsa-miR-1-3p:MIMAT0000416 | 132.0 | 0.0001 |
| hsa-miR-16-5p:MIMAT0000069 | 10.4 | 0.0002 |
| hsa-mir-378c:MI0015825 | 0.039 | 0.0002 |
| hsa-miR-146a-5p:MIMAT0000449 | 12.2 | 0.0003 |
| hsa-miR-378d:MIMAT0018926 | 0.042 | 0.0004 |
| hsa-miR-532-5p:MIMAT0002888 | 8.0 | 0.0005 |
| hsa-miR-191-5p:MIMAT0000440 | 6.5 | 0.0015 |
| hsa-miR-221-3p:MIMAT0000278 | 10.2 | 0.0015 |
| hsa-miR-328-3p:MIMAT0000752 | 0.057 | 0.0015 |
| hsa-let-7f-5p:MIMAT0000067 | 4.9 | 0.014 |
| hsa-let-7e-5p:MIMAT0000066 | 0.164 | 0.048 |
| hsa-miR-10a-5p:MIMAT0000253 | 4.2 | 0.048 |

**Supplemental Table 7: microRNA (miRNA) to messenger (mRNA) targets for hub genes identified on Weighted gene co-expression network analysis (WGCNA).**

| ID | Symbol | ID | Symbol | ID | Symbol |
| --- | --- | --- | --- | --- | --- |
| hsa-let-7f-5p | ABCB9 | **hsa-let-7f-5p** | EIF4G2 | **hsa-let-7f-5p** | MTRR |
| hsa-let-7f-5p | ADAMTS14 | **hsa-let-7f-5p** | ELP1 | **hsa-let-7f-5p** | MXD1 |
| hsa-let-7f-5p | ADAMTS8 | **hsa-let-7f-5p** | F2 | **hsa-let-7f-5p** | MYC |
| hsa-let-7f-5p | ADGRG1 | **hsa-let-7f-5p** | FADS2 | **hsa-let-7f-5p** | MYO1F |
| hsa-let-7f-5p | ADRB2 | **hsa-let-7f-5p** | FANCD2 | **hsa-let-7f-5p** | NDST2 |
| hsa-let-7f-5p | ADRB3 | **hsa-let-7f-5p** | FASLG | **hsa-let-7f-5p** | NEDD4 |
| hsa-let-7f-5p | AGBL2 | **hsa-let-7f-5p** | FOXP2 | **hsa-let-7f-5p** | NF2 |
| hsa-let-7f-5p | AIFM1 | **hsa-let-7f-5p** | FZD3 | **hsa-let-7f-5p** | NGF |
| hsa-let-7f-5p | AP1S1 | **hsa-let-7f-5p** | GALNT1 | **hsa-let-7f-5p** | NID1 |
| hsa-let-7f-5p | APBB3 | **hsa-let-7f-5p** | GAN | **hsa-let-7f-5p** | NME4 |
| hsa-let-7f-5p | ATP6V0A1 | **hsa-let-7f-5p** | GAS7 | **hsa-let-7f-5p** | NPHP3 |
| hsa-let-7f-5p | AURKB | **hsa-let-7f-5p** | GIPC1 | **hsa-let-7f-5p** | NR6A1 |
| hsa-let-7f-5p | AVEN | **hsa-let-7f-5p** | GLRX | **hsa-let-7f-5p** | ONECUT2 |
| hsa-let-7f-5p | B4GAT1 | **hsa-let-7f-5p** | GNPTAB | **hsa-let-7f-5p** | P3H1 |
| hsa-let-7f-5p | BACH1 | **hsa-let-7f-5p** | GPR157 | **hsa-let-7f-5p** | P4HA2 |
| hsa-let-7f-5p | BCL2L1 | **hsa-let-7f-5p** | GPR63 | **hsa-let-7f-5p** | PAPPA |
| hsa-let-7f-5p | BEGAIN | **hsa-let-7f-5p** | GRID2IP | **hsa-let-7f-5p** | PBX1 |
| hsa-let-7f-5p | BSG | **hsa-let-7f-5p** | GRIN2B | **hsa-let-7f-5p** | PDGFA |
| hsa-let-7f-5p | CALM1 | **hsa-let-7f-5p** | GTPBP3 | **hsa-let-7f-5p** | PDGFB |
| hsa-let-7f-5p | CARHSP1 | **hsa-let-7f-5p** | HAND1 | **hsa-let-7f-5p** | PEX11B |
| hsa-let-7f-5p | CASP3 | **hsa-let-7f-5p** | HIF3A | **hsa-let-7f-5p** | PGRMC1 |
| hsa-let-7f-5p | CBFA2T3 | **hsa-let-7f-5p** | HMGA2 | **hsa-let-7f-5p** | PLD3 |
| hsa-let-7f-5p | CCL3 | **hsa-let-7f-5p** | HMOX1 | **hsa-let-7f-5p** | PLEC |
| hsa-let-7f-5p | CCND1 | **hsa-let-7f-5p** | HOXA1 | **hsa-let-7f-5p** | PLEKHO1 |
| hsa-let-7f-5p | CCND2 | **hsa-let-7f-5p** | HOXA9 | **hsa-let-7f-5p** | PLOD2 |
| hsa-let-7f-5p | CCNF | **hsa-let-7f-5p** | HOXB1 | **hsa-let-7f-5p** | POLR2D |
| hsa-let-7f-5p | CCR7 | **hsa-let-7f-5p** | ICMT | **hsa-let-7f-5p** | POU2F1 |
| hsa-let-7f-5p | CDIPT | **hsa-let-7f-5p** | IFRD1 | **hsa-let-7f-5p** | POU2F2 |
| hsa-let-7f-5p | CDKN1A | **hsa-let-7f-5p** | IGDCC3 | **hsa-let-7f-5p** | PPARGC1B |
| hsa-let-7f-5p | CHRNA7 | **hsa-let-7f-5p** | IGF1R | **hsa-let-7f-5p** | PPP1R7 |
| hsa-let-7f-5p | CLCN5 | **hsa-let-7f-5p** | IL10 | **hsa-let-7f-5p** | PRKAR2A |
| hsa-let-7f-5p | COL1A1 | **hsa-let-7f-5p** | ITGB3 | **hsa-let-7f-5p** | PTGS2 |
| hsa-let-7f-5p | COL1A2 | **hsa-let-7f-5p** | KCNJ11 | **hsa-let-7f-5p** | QARS1 |
| hsa-let-7f-5p | COL5A1 | **hsa-let-7f-5p** | KCNJ16 | **hsa-let-7f-5p** | RAB15 |
| hsa-let-7f-5p | CPEB1 | **hsa-let-7f-5p** | KPNA5 | **hsa-let-7f-5p** | RDX |
| hsa-let-7f-5p | CYP19A1 | **hsa-let-7f-5p** | KRAS | **hsa-let-7f-5p** | RHOB |
| hsa-let-7f-5p | DIAPH2 | **hsa-let-7f-5p** | KRT19 | **hsa-let-7f-5p** | S100A8 |
| hsa-let-7f-5p | DPH3 | **hsa-let-7f-5p** | LPGAT1 | **hsa-let-7f-5p** | SALL4 |
| hsa-let-7f-5p | DRD3 | **hsa-let-7f-5p** | MAP3K13 | **hsa-let-7f-5p** | SCN11A |
| hsa-let-7f-5p | DSP | **hsa-let-7f-5p** | MAP4K3 | **hsa-let-7f-5p** | SCN4B |
| hsa-let-7f-5p | DUSP12 | **hsa-let-7f-5p** | MDM4 | **hsa-let-7f-5p** | SCN8A |
| hsa-let-7f-5p | DUSP16 | **hsa-let-7f-5p** | MFSD4A | **hsa-let-7f-5p** | SEC16B |
| hsa-let-7f-5p | DUSP22 | **hsa-let-7f-5p** | MIB1 | **hsa-let-7f-5p** | SEPTIN3 |
| hsa-let-7f-5p | DYNC2LI1 | **hsa-let-7f-5p** | MTPN | **hsa-let-7f-5p** | SIGMAR1 |
| ID | **Symbol** | **ID** | **Symbol** | **ID** | **Symbol** |
| hsa-let-7f-5p | SLC1A4 | **hsa-miR-146a-5p** | CCNA2 | **hsa-miR-146a-5p** | TRAF6 |
| hsa-let-7f-5p | SKIL | **hsa-miR-146a-5p** | CCNI | **hsa-miR-146a-5p** | TTPA |
| hsa-let-7f-5p | SLC12A9 | **hsa-miR-146a-5p** | CCR3 |  |  |
| hsa-let-7f-5p | SLC20A1 | **hsa-miR-146a-5p** | CDKN3 |  |  |
| hsa-let-7f-5p | SLC25A18 | **hsa-miR-146a-5p** | CHUK |  |  |
| hsa-let-7f-5p | SLC38A1 | **hsa-miR-146a-5p** | CXCL8 |  |  |
| hsa-let-7f-5p | SLX1B | **hsa-miR-146a-5p** | CXCR4 |  |  |
| hsa-let-7f-5p | SMARCC1 | **hsa-miR-146a-5p** | DTNA |  |  |
| hsa-let-7f-5p | SMIM3 | **hsa-miR-146a-5p** | FBXW2 |  |  |
| hsa-let-7f-5p | SNAP23 | **hsa-miR-146a-5p** | FZD3 |  |  |
| hsa-let-7f-5p | SOCS1 | **hsa-miR-146a-5p** | HDAC8 |  |  |
| hsa-let-7f-5p | SPARC | **hsa-miR-146a-5p** | HSPA1A |  |  |
| hsa-let-7f-5p | SSH1 | **hsa-miR-146a-5p** | IL10 |  |  |
| hsa-let-7f-5p | STARD4 | **hsa-miR-146a-5p** | IL12RB2 |  |  |
| hsa-let-7f-5p | STK40 | **hsa-miR-146a-5p** | IL1R1 |  |  |
| hsa-let-7f-5p | STX3 | **hsa-miR-146a-5p** | IL1RAP |  |  |
| hsa-let-7f-5p | TARBP2 | **hsa-miR-146a-5p** | IRAK1 |  |  |
| hsa-let-7f-5p | TGFBR1 | **hsa-miR-146a-5p** | IRAK2 |  |  |
| hsa-let-7f-5p | TGFBR2 | **hsa-miR-146a-5p** | IRF5 |  |  |
| hsa-let-7f-5p | TGFBR3 | **hsa-miR-146a-5p** | LBP |  |  |
| hsa-let-7f-5p | THRA | **hsa-miR-146a-5p** | LTF |  |  |
| hsa-let-7f-5p | THRSP | **hsa-miR-146a-5p** | MCPH1 |  |  |
| hsa-let-7f-5p | TLR4 | **hsa-miR-146a-5p** | MMP16 |  |  |
| hsa-let-7f-5p | TMEM178B | **hsa-miR-146a-5p** | MR1 |  |  |
| hsa-let-7f-5p | TP53 | **hsa-miR-146a-5p** | NFIX |  |  |
| hsa-let-7f-5p | TPM1 | **hsa-miR-146a-5p** | NLGN1 |  |  |
| hsa-let-7f-5p | TTLL4 | **hsa-miR-146a-5p** | NOS2 |  |  |
| hsa-let-7f-5p | TUBB4A | **hsa-miR-146a-5p** | NOVA1 |  |  |
| hsa-let-7f-5p | TUSC2 | **hsa-miR-146a-5p** | NUMB |  |  |
| hsa-let-7f-5p | UGCG | **hsa-miR-146a-5p** | PDGFRA |  |  |
| hsa-let-7f-5p | UGT8 | **hsa-miR-146a-5p** | PGLYRP1 |  |  |
| hsa-let-7f-5p | UTRN | **hsa-miR-146a-5p** | PPM1K |  |  |
| hsa-let-7f-5p | VCAN | **hsa-miR-146a-5p** | PRDX4 |  |  |
| hsa-let-7f-5p | VIM | **hsa-miR-146a-5p** | PRKAA2 |  |  |
| hsa-let-7f-5p | VPS39 | **hsa-miR-146a-5p** | PTGES2 |  |  |
| hsa-let-7f-5p | VSTM5 | **hsa-miR-146a-5p** | SDCBP2 |  |  |
| hsa-let-7f-5p | WNT1 | **hsa-miR-146a-5p** | SFTPD |  |  |
| hsa-let-7f-5p | XCR1 | **hsa-miR-146a-5p** | SLC1A1 |  |  |
| hsa-miR-146a-5p | APPL1 | **hsa-miR-146a-5p** | STAT1 |  |  |
| hsa-miR-146a-5p | ARID1A | **hsa-miR-146a-5p** | SYT1 |  |  |
| hsa-miR-146a-5p | BLMH | **hsa-miR-146a-5p** | TGIF1 |  |  |
| hsa-miR-146a-5p | BRCA1 | **hsa-miR-146a-5p** | TIMELESS |  |  |
| hsa-miR-146a-5p | BSG | **hsa-miR-146a-5p** | TLR1 |  |  |
| hsa-miR-146a-5p | CAMP | **hsa-miR-146a-5p** | TLR10 |  |  |
| hsa-miR-146a-5p | CASP7 | **hsa-miR-146a-5p** | TLR4 |  |  |
| hsa-miR-146a-5p | CCK | **hsa-miR-146a-5p** | TLR9 |  |  |
